# Inhibition of cGAS-STING by JQ1 alleviates oxidative stress-induced retina inflammation and degeneration

**DOI:** 10.1038/s41418-022-00967-4

**Published:** 2022-03-28

**Authors:** Ming Zou, Qin Ke, Qian Nie, Ruili Qi, Xingfei Zhu, Wei Liu, Xuebin Hu, Qian Sun, Jia-Ling Fu, Xiangcheng Tang, Yizhi Liu, David Wan-Cheng Li, Lili Gong

**Affiliations:** State Key Laboratory of Ophthalmology, Zhongshan Ophthalmic Center, Sun Yat-Sen University, Guangzhou, 510060 Guangdong China

**Keywords:** Epigenetics, Drug development

## Abstract

Atrophic (“dry”) form of age-related macular degeneration (AMD) is a leading cause of vision loss characterized by macular retinal pigment epithelium (RPE) and the ensuing photoreceptor degeneration. cGAS-STING signaling is a key cytosolic DNA sensor system in innate immunity and have recently been shown promotes RPE degeneration. However, expression regulation and therapeutic potential of cGAS and STING are not explored in retina under dry AMD pathogenic conditions. Our analysis shows upregulated *STING* RNA and increased chromatin accessibility around *cGAS* and *STING* promoters in macular retinas from dry AMD patients. cGAS-STING activation was detected in oxidative stress-induced mouse retina degeneration, accompanied with cytosolic leakage of damaged DNA in photoreceptors. Pharmaceutical or genetic approaches indicates STING promotes retina inflammation and degeneration upon oxidative damage. Drug screening reveals that BRD4 inhibitor JQ1 reduces cGAS-STING activation, inflammation and photoreceptor degeneration in the injured retina. BRD4 inhibition epigenetically suppresses *STING* transcription, and promotes autophagy-dependent cytosolic DNA clearance. Together, our results show that activation of cGAS-STING in retina may present pivotal innate immunity response in GA pathogenesis, whereas inhibition of cGAS-STING signaling by JQ1 could serve as a potential therapeutic strategy.

## Abstract (web summary)

Schematic summary of the mechanism underlying BRD4 inhibition on cGAS-STING signaling during retina degeneration. Cytosolic DNA accumulation and activation of cGAS-STING pathway were detected in retina photoreceptors after oxidative injury. BRD4 inhibition alleviates retinal inflammation and degeneration by epigenetically silencing *STING* transcription and by promoting autophagy-dependent cytosolic DNA clearance.

## Introduction

Age-related macular degeneration (AMD) is the most prevalent blind-causing eye disease in the elderly. Currently no approved treatment exist for the nonvascular or dry AMD, which account for 90% of all AMD patients [1]. AMD is initiated by progressive degeneration of the retinal pigment epithelium (RPE), and results in permanent visual loss caused by photoreceptor death in the macular region [2]. Recent foundlings demonstrated dysfunction of innate immunity in disease pathogenesis, hence immunomodulation is emerging as a promising strategy for dry AMD treatment [3–6].

Cyclic GMP-AMP Synthase-Stimulator of Interferon Genes (cGAS-STING) signaling is a key innate immune response detecting cytosolic DNA [7, 8]. Activated STING induces production of type I interferons via IRF3/IRF7 and other inflammatory cytokines via the NFκB pathway, respectively. [9–11]. Gain-of-function mutations in STING cause severe auto-inflammatory diseases [12], whereas inhibition of cGAS-STING signaling is beneficial in diverse inflammatory injuries [13–15]. Recently, elevated cGAS level was detected in the RPE of geographic atrophy (GA), an advanced form of dry AMD, and causally linked to RPE degeneration [16], highlighting the importance of cGAS-STING signaling in dry AMD.

DNA from oxidative stress (OS)-exposed cell potently triggers STING activation and led to enhanced immune recognition [17]. The cross-talk between OS and inflammation have an intimate effect in RPE and retina degeneration [18, 19]. However, OS-mediated DNA damage and cytosolic leakage, and related retina inflammation remains elusive. Moreover, drugs promoting cytosolic DNA clearance may help to inhibit unwanted innate immune response during retina degeneration.

BRD4, a member of Bromodomain and extraterminal domain (BET) proteins, plays pivotal roles in inflammation through assembly of acetylated histone and acetylation-dependent chromatin complexes on inflammatory genes [20, 21]. Our analysis reveals that BRD4 inhibition reduces active chromatin mark as well as Polymerase II (Pol II) to *STING* in mouse bone marrow-derived macrophages. However, whether and how BRD4 inhibition represses *STING* transcription in the context of AMD pathogenesis is unknown. On the other side, BRD4 has been recently shown represses autophagy gene expression, and BRD4 inhibition selectively activates protein aggregates-induced aggrephagy [22]. The effects of BRD4 on cytosolic DNA autophagy remain elusive.

Here, we demonstrated activation of cGAS-STING promotes OS-induced retina degeneration and inflammation. BRD4 was induced by OS, and BRD4 inhibition suppresses cGAS-STING signaling and alleviates retina degeneration upon oxidative injury.

## Results

### *STING* is elevated in macular retina of dry AMD patients and cGAS-STING signaling is activated in mouse retina upon oxidative injury

**Fig. 1:**
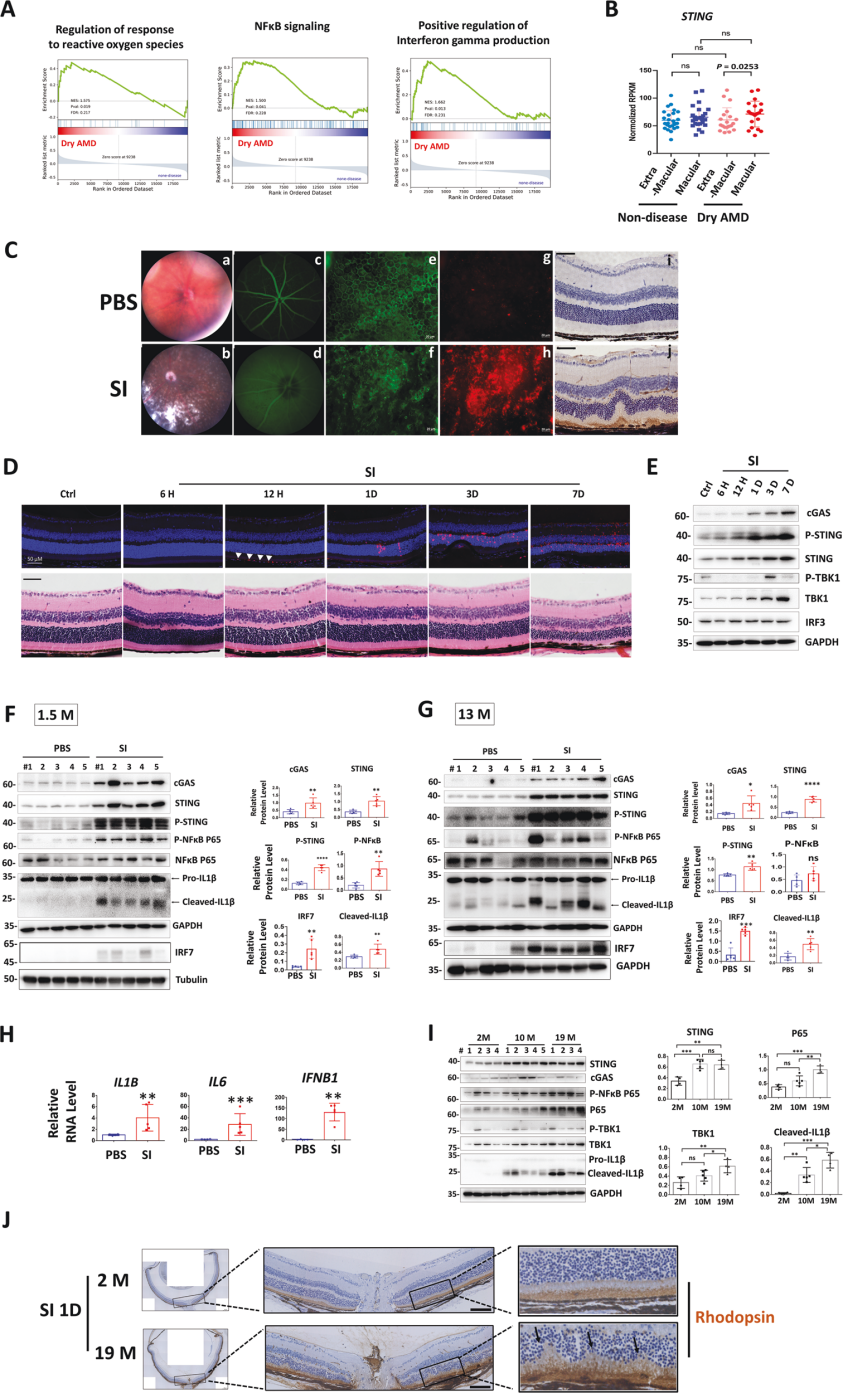
*STING* is elevated in macular retina of dry AMD patients and cGAS-STING signaling is activated during mouse retina degeneration. **A** Gene set enrichment analysis (GSEA) profiles showing significant enrichment of gene sets associated with indicated pathway in dry AMD retinas (*n* = 41) compared to normal retinas (*n* = 55) (GSE29801) [24]. The false discovery rate (FDR) < 0.25 for pathways mentioned. **B** RNA-seq analysis of *STING* expression in extra-macular and macular retinas of normal (*n* = 26) and dry AMD patients (*n* = 20) [24]. Comparisons were made between retinas from normal and dry AMD patients (unpaired *t*-test), and the extra-macular and macular retinas of the same eye (paired *t*-test). **C**–**J** Mice were intraperitoneal (IP) injected with PBS or sodium iodate (SI) (35 mg/kg) and analysis was conducted 3-day post injection otherwise indicated. **C** Fundus photography (a, b) and fluorescein angiography (c, d) showing eye morphology. RPE flat mounts stained with phallodin-FITC to label the F-actin (e, f) and with IBA1 antibody to label the mononuclear phagocytes (MP) (g, h). Scale bar: 20 μM. (*n* = 3). (i, j) Immunohistochemistry (IHC) showing IBA1-positive MP in retina sections. Scale bar: 50 μM (*n* = 2). **D** Upper panels: Terminal deoxynucleotidyl transferase dUTP nick end labeling (TUNEL) analysis showing cell death. Arrow head: TUNEL-positive RPE cells. Lower panels: Hematoxylin and eosin (HE) staining. (*n* = 3) H: hour, D: day. Scale bar: 50 μM. Western blot (WB) analysis showing retina proteins at the indicated time (**E**) or 3-day (**F**, **G**) post injection. Right panels: quantification results of WB. M month. **H** qRT-PCR analysis of retina RNA 3-day post injection. **I** WB showing retina proteins. Right panels: quantification results of WB. **J** IHC analysis using rhodopsin antibody shows retina structure 1-day post SI injection. Arrows indicated destruction of photoreceptors. (*n* = 3). Scale bar:100 μM.

Our recent analysis showed that STING was upregulated in human macular RPE as compared with extra-macular, although healthy controls and GA patients exhibit similar trend [23]. Interestingly, analysis of retina transcriptomes [24] reveals enrichment of genes involved in the interferon gamma pathway, NFκB signaling and regulation of reactive oxygen species (ROS) (Fig. 1A). Further, *STING* was specifically increased in retina macular of dry AMD patients (Fig. 1B). Next, cGAS-STING signaling was investigated in an acute RPE and retina degeneration mouse model induced by oxidant sodium iodate (SI). Consistent with previous reports [25–28], SI led to primary RPE degeneration and cell death, followed by secondary photoreceptor death, and accumulation of mononuclear phagocytes (MPs) in the subretinal space and retina (Fig. 1C, D), resembling GA pathogenesis [26, 29, 30]. Notably, we detected time-dependent upregulation of cGAS, STING, TBK1 and phosphorylated STING in retinas that was correlated with retinal cell damage (Fig. 1E). Since prominent photoreceptor death occurred at 3 days post SI injection (Fig. 1D), we expand our investigation at this time point and detected activation of cGAS-STING signaling in both young (1.5-month) and middle-aged (13-month) mouse retinas (Fig. 1F–H). Notably, IRF7 but not IRF3 was activated upon SI injury (Fig. S1). Importantly, during physiological aging, STING was already increased in middle-aged mouse retinas, while p65, TBK1 and active IL1β were upregulated in an age-dependent manner (Fig. 1I). In addition, old mice showed enhanced sensitivity to SI injury (Fig. 1J). Together, cGAS-STING signaling was activated in OS-induced retina degeneration and retina aging.

### STING promotes retina inflammation upon oxidative injury

**Fig. 2:**
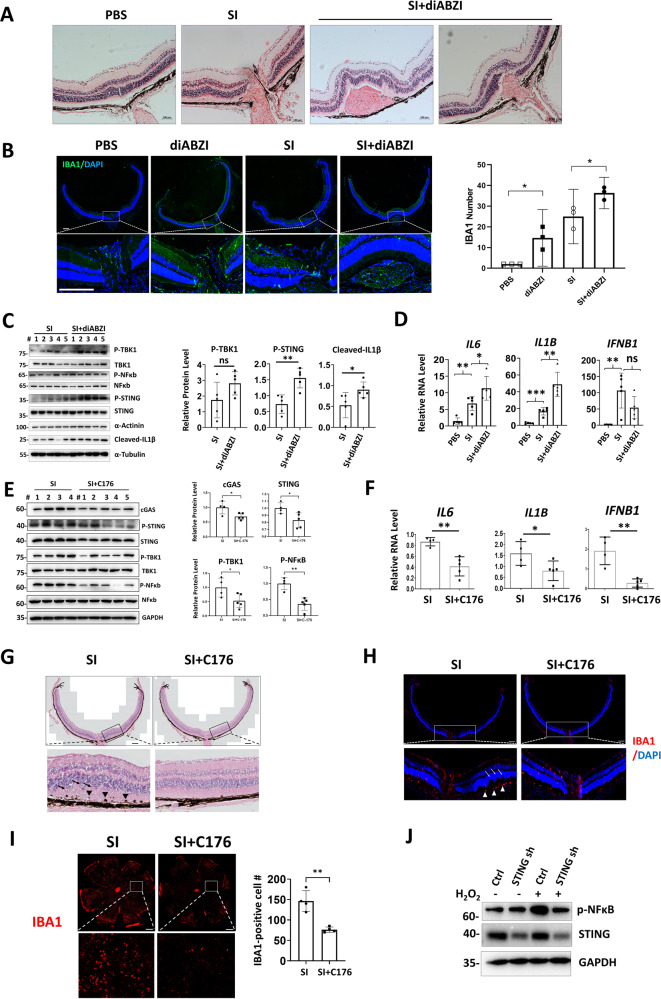
STING promotes retina inflammation upon oxidative injury. **A** HE staining of mouse retina. Subretinal bleeding was observed in retinas from 4 out of 6 diABZI-injected mice. Scale bar: 100 μM. **B** IF analysis showing IBA1-positive MPs. Note that diABZI alone increased MP infiltration around the optic nerve. Right panel: quantification of IBA1-positive cells. Scale bar: 200 μM. WB (**C**) or qRT-PCR (**D**) showing indicated retinal proteins or gene expression. WB (**E**) or qRT-PCR (**F**) showing indicated retinal proteins or gene expression. **G** HE staining of mouse retina. Arrows indicate retina disorganization and arrow heads indicate swelling and bundling of RPE cells. Scale bar: 200 μM. **H** IF analysis showing IBA1-positive MPs in retina. Arrows: destructive retina, arrow heads: subretinal localization of MPs. *n* = 4. Scale bar: 200 μM. **I** IF analysis shows IBA1-positive MPs in RPE flat mounts. *n* = 4. Scale bar: 500 μM. Right panel: IBA1-positive cell number in four randomly selected regions. **J** WB showing indicated proteins in ARPE-19 cells. Cells were exposed to 600 μM H_2_O_2_ for 2 h and recovered for 2 h before analysis.

Because STING was increased in AMD retina, we determined the effects of STING on retina inflammation upon SI injury. Injection of diABZI (dimeric amidobenzimidazole), a STING agonist that binds to the cGAMP binding pocket of STING [31], led to retinal bleeding and infiltration of MPs when combined with SI (Fig. 2A, B), and diABZI treatment alone increased immune cell accumulation around the optic nerve (Fig. 2B). Further, combined injection of diABZI and SI augments cGAS-STING activation (Fig. 2C, D). In contrast, C176, a STING inhibitor preventing STING palmitoylation [32], reduced cGAS-STING activation after SI exposure (Fig. 2E, F). In addition, C176 suppressed SI-induced retina destruction and subretinal immune cell infiltration (Fig. 2G–I). Finally, depletion of STING decreased OS-induced NFκB activation in RPE cells (Fig. 2J). Therefore, STING promotes OS-induced retinal inflammation and NFκB activation.

### OS-induced DNA damage and cytosolic leakage provoke retinal cell inflammation

**Fig. 3:**
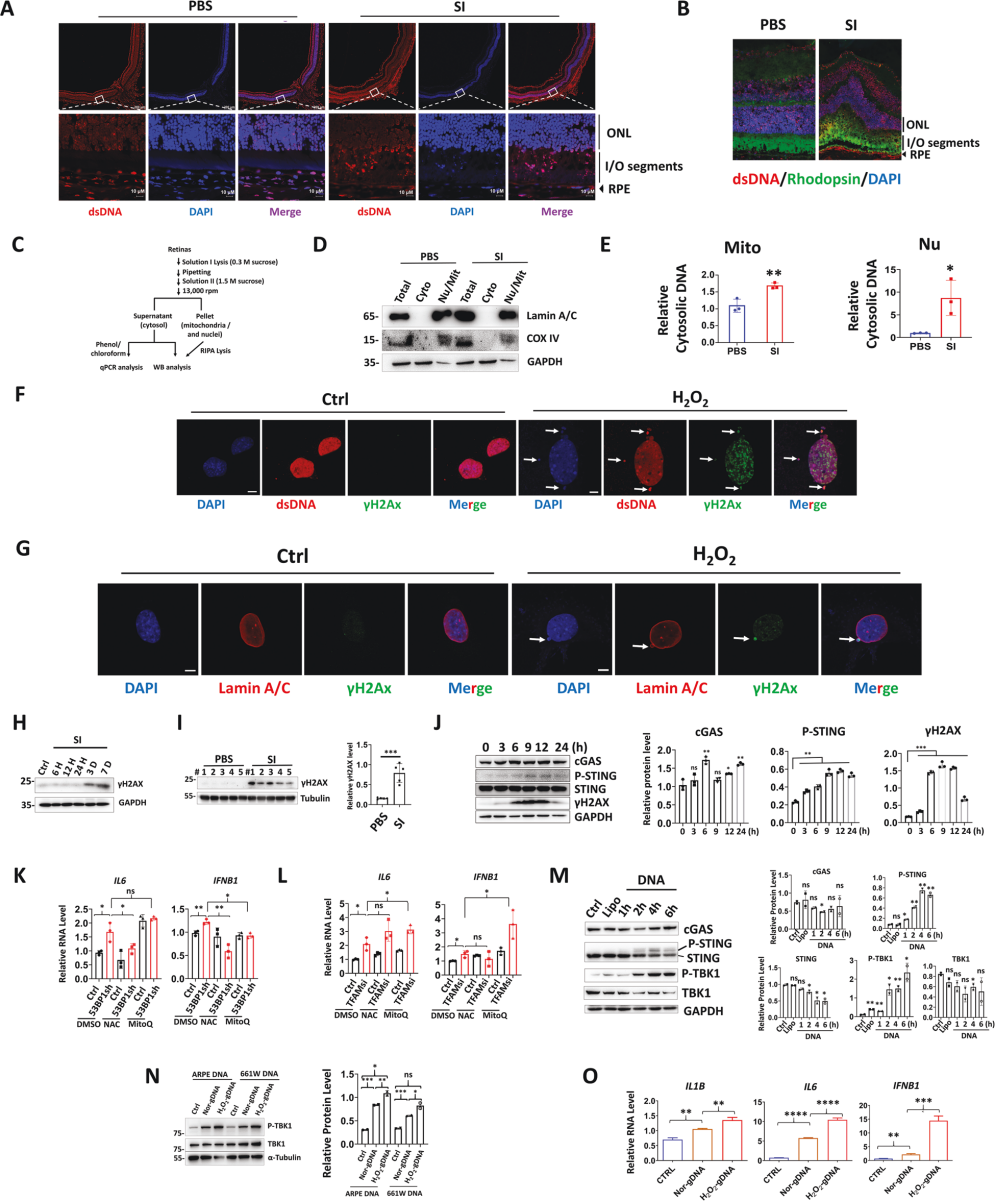
OS-induced DNA damage and cytosolic leakage provoke retinal cell inflammation. Immunofluorescence (IF) staining for dsDNA **A** and dsDNA/rhodopsin **B** in retina. The DNA was counterstained with DAPI. Scale bar: 200 μM. **C** Schematic diagram for preparation of nuclei- and mitochondria-free retinal cytosol. **D** WB showing separation of retina nuclear and cytosolic fractions. **E** q-PCR analysis of cytosolic nuclear and mitochondrial (Mito) DNA. **F**, **G** IF staining in 661W cells using the indicated antibodies. Arrows: cytosolic DNA with positive γH2Ax labeling. Scale bar: 5 μM. WB showing retina proteins at the indicated time points (**H**) or 3-day (**I**) post SI injection. **J** WB showing indicated proteins in 661W cells. Cells were treated with or without Ara-C (10 μM) and collected at the indicated time points. **K**, **L** qRT-PCR analysis showing gene expression in ARPE cells. Cells were treated with DMSO or NAC (1 mM) or MitoQ (1 μM) for 24 h before collection. **M**, **N** WB analysis showing protein expression. **M** 661W cells were untreated (Ctrl) or, transfected with lipofectamine or linearized GFP-C3 plasmid (1 ug/ml) and harvested at the indicated time points. **N** ARPE cells were transfected with lipofectamine (Ctrl), or genomic DNA isolated from normal (nor-gDNA) or H_2_O_2_-exposred (H_2_O_2_-gDNA) ARPE or 661W cells (2 ug/ml) and collected 16 h post transfection. **O** qRT-PCR analysis showing indicated genes in ARPE cells transfected with or without ARPE genomic DNA as described as Fig. 6N.

cGAS-STING is a sensor system of cytosolic DNA, therefore, we investigated the presence of double-stranded DNA (dsDNA) in control and injured retina. Immunofluorescence (IF) analysis showed SI injury led to prominent cytosolic dsDNAs leakage in the inner and outer segments of rods and cones (Fig. 3A, B). To further delineate the source of cytosolic DNA, the cytosol was separated from nuclear and mitochondria fractions in the control or damaged retinas (Fig. 3C, D). Quantitative real-time PCR (qPCR) revealed that SI-induced cytosolic DNA was derived from both mitochondrial and nuclear, with nuclei showing more prominent leakage (Fig. 3E). Consistently, OS leads to leakage of nuclear DNA into cytosol and formation of micronuclei in mouse photoreceptor cell line 661W (Fig. 3F, arrows). Furthermore, cytosolic DNA are positive for DNA damage marker γH2AX, possible by extrusion of chromatin through nuclear envelope (Fig. 3G, arrows). Western blot (WB) analysis confirmed existence of DNA damage in retinas after SI exposure (Fig. 3H, I), or in 661W cells upon treatment of genotoxic replication inhibitor cytosine β-D-arabinofuranoside hydrochloride (Ara-C) (Fig. 3J), a drug known to result in cytosolic DNA accumulation [33]. To delineate relationship between cytosolic DNA and inflammation, 53BP1 or mitochondrial transcription factor A (TFAM) were knocked down to induce nuclear or mitochondrial DNA leakage, respectively [34, 35] (Fig. S2). IL6 and IFNβ was induced in both knockdown conditions, while addition of antioxidant N-Acetylcysteine (NAC) repressed such induction with 53BP1 but not TFAM knockdown. MitoQ, the antioxidant for mitochondrial DNA damage, was largely ineffective in IL6 and IFNβ repression (Fig. 3K, L). Finally, direct transfection of DNA in cytosol activates STING and the downstream TBK1 (Fig. 3M), and H_2_O_2_-treated cellular DNA triggers stronger TBK1 activation and inflammatory factor production, while the cellular source of genomic DNA is irrelevant for TBK1 activation (Fig. 3N, O). Together, these results show OS-induced cytosolic leakage of damaged DNA activates cGAS-STING signaling in retinal cells.

### BRD4 inhibitors repress STING expression and STING-mediated NFκB activation

**Fig. 4:**
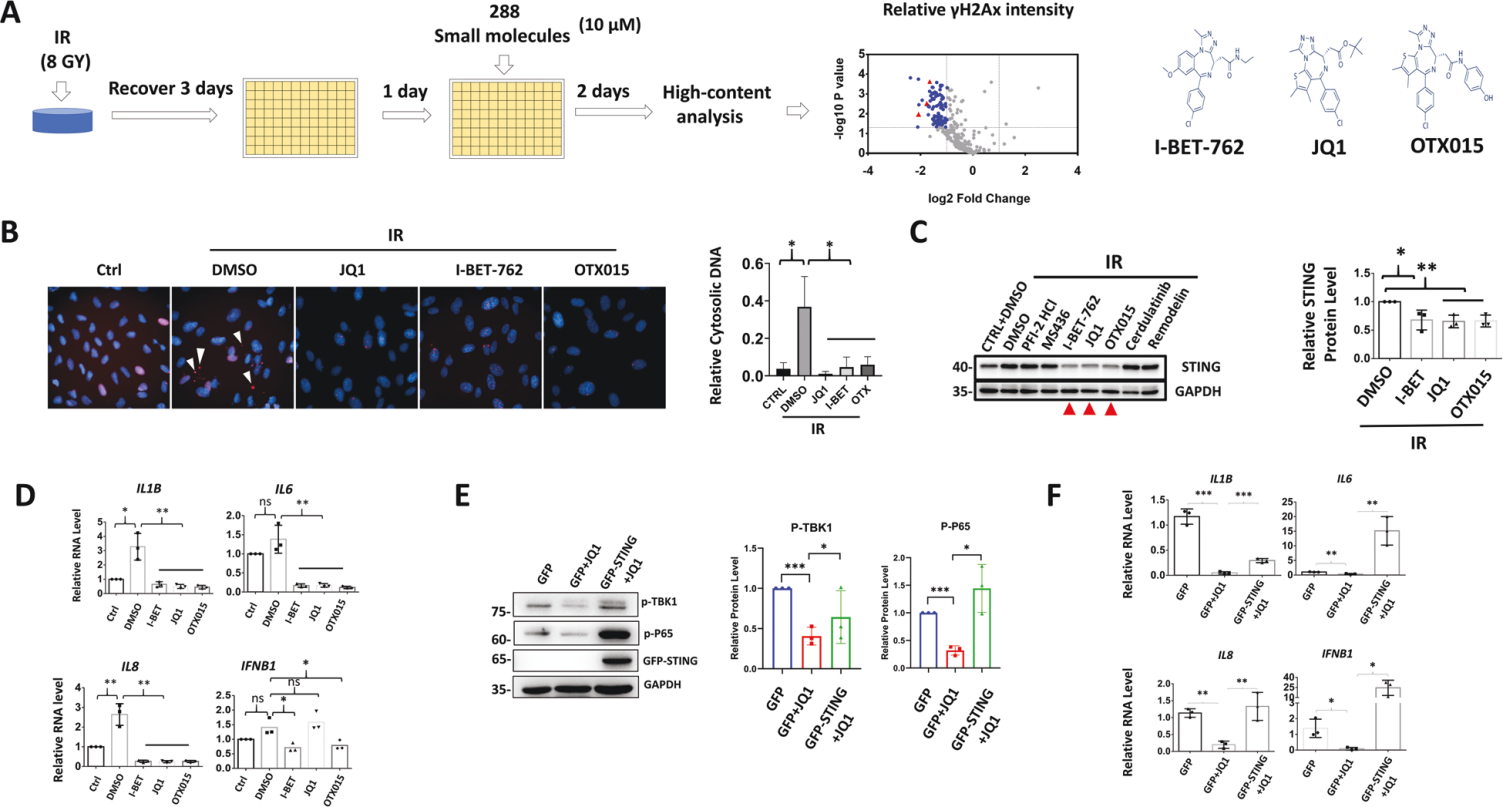
BRD4 inhibitors repress STING expression and STING-mediated NFκB activation. **A** Schematic diagram shows drug screening process. The red triangles represent identified BRD4 inhibitors. **B** Representative images from high-content analysis. Arrow heads: γH2Ax-positive cytosolic DNA. Right panel: relative cytosolic DNA number after normalized to nuclei numbers, 30–50 cells counted. WB or qRT-PCR analysis showing protein **C** or gene **D** expression in ARPE-19 cells. Selection of other small molecules in WB was based on results from high-content analysis. Treatment conducted as described in **A**. WB **E** or qRT-PCR **F** analysis conducted in 661W treated with or without 10 μM of JQ1 for 24 h.

Next, we sought to determine small molecules that can inhibit DNA damage and cytosolic DNA accumulation. Here, high-content analysis of the fluorescence intensity of γH2Ax as a readout was used to screen an epigenetic drug library (Fig. 4A). BRD4 inhibitors, I-BET-762 (I-BET), JQ1 and OTX015 (OTX) significantly decreased γH2Ax signals and cytosolic DNA leakage after X-ray irradiation (IR) (Fig. 4B). Notably, only the BRD4 inhibitors and no other small molecules repressing γH2Ax reduced STING levels after IR (Fig. 4C). BRD4 inhibitors also suppressed expression of downstream inflammatory factors (Fig. 4D). Finally, STING overexpression reversed the repressive effects of JQ1 on NFκB and TBK1 activation (Fig. 4E), and on inflammatory factor production (Fig. 4F). Together, BRD4 inhibitors reduce cytosolic DNA and suppress NFκB signaling in a STING-dependent manner.

### BRD4 inhibitors epigenetically represses *STING* transcription

**Fig. 5:**
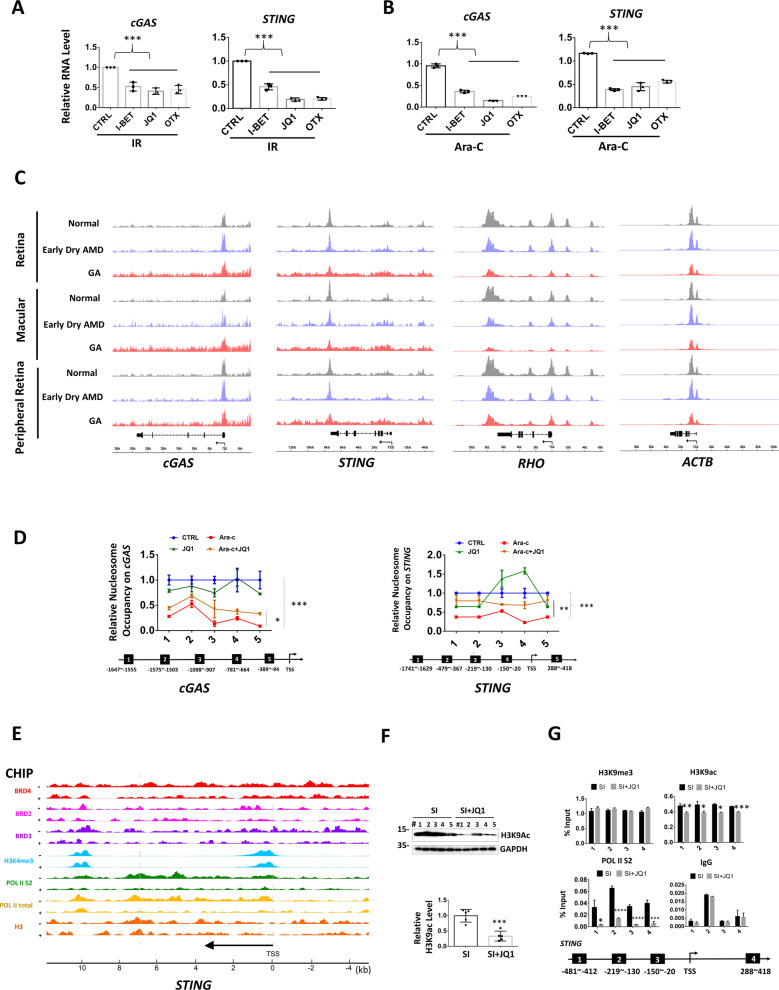
BRD4 inhibitors epigenetically represses *STING* transcription. **A**, **B** qRT-PCR analysis for gene expression. The detailed treatment was described in “Methods” section. **C** ATAC-Seq shows chromatin accessibility in the indicated genes. (macular: *n* = 5 for normal, *n* = 2 for early dry AMD, *n* = 5 for GA; peripheral macular: *n* = 6 for normal, *n* = 4 for early dry AMD, *n* = 4 for GA) [37]. **D** Micrococcal nuclease digestion assay shows chromatin compaction in 661W cells. Treatment was the same in **B**. TSS transcription start site. **E** Epigenetic profiles of *STING* in mouse macrophage treated with 5 μM of I-BET762 (+) or DMSO (−). Original data from published ChIP-Seq [39]. The *y*-axes represent the average number of tags per gene per 25 base pairs per 1,000,000 mapped reads. Scale values are indicated in parentheses. **F** WB analysis of retina proteins in the indicated treatment. **G** ChIP assay shows association of the indicated proteins with the *STING*.

BRD4 inhibitors reduced cGAS and STING mRNA after IR or Ara-C treatment (Fig. 5A, B). As epigenetic modulator, BRD4 activates gene transcription through nucleosomes eviction and chromatin decompaction [36]. Intriguing, after analyzing the public ATAC-seq data [37], we found both *cGAS* and *STING* promoters showed increased chromatin accessibility in retinas from GA patients, especially in the macular region, which is in great contrast to the reported decreased chromatin accessibility here (*rhodopsin* and *actin*, for example) (Fig. 5C). Nucleosome occupancy analysis showed JQ1 significantly reversed Ara-C-induced chromatin opening at *cGAS* and *STING* promoters (Fig. 5D), suggesting JQ1 epigenetically represses cGAS and STING. BRD4 links histone acetylation to transcription, leading to increased RNA Pol II phosphorylation and transcription [38, 39]. We thus analyzed chromatin immunoprecipitation sequencing data from published datasets with or without BRD4 inhibitor I-BET treatment [39]. I-BET led to marked reduction of BRD4 enrichment and completely diminished Pol II and Pol II S2 association on *STING* gene (Fig. 5E). In retina, JQ1 effectively inhibited acetylation of H3K9 (Fig. 5F), and reduced H3K9Ac and phosphorylated Pol II enrichment on *STING* without altering heterochromatin mark H3K9me3 (Fig. 5G). Taken together, these results showed *cGAS* and *STING* promoters were more accessible in the macular retinas of GA patients, while JQ1 silenced *STING* through decreasing chromatin accessibility and Pol II recruitment.

### OS-induced BRD4 promotes STING expression and inflammation

**Fig. 6:**
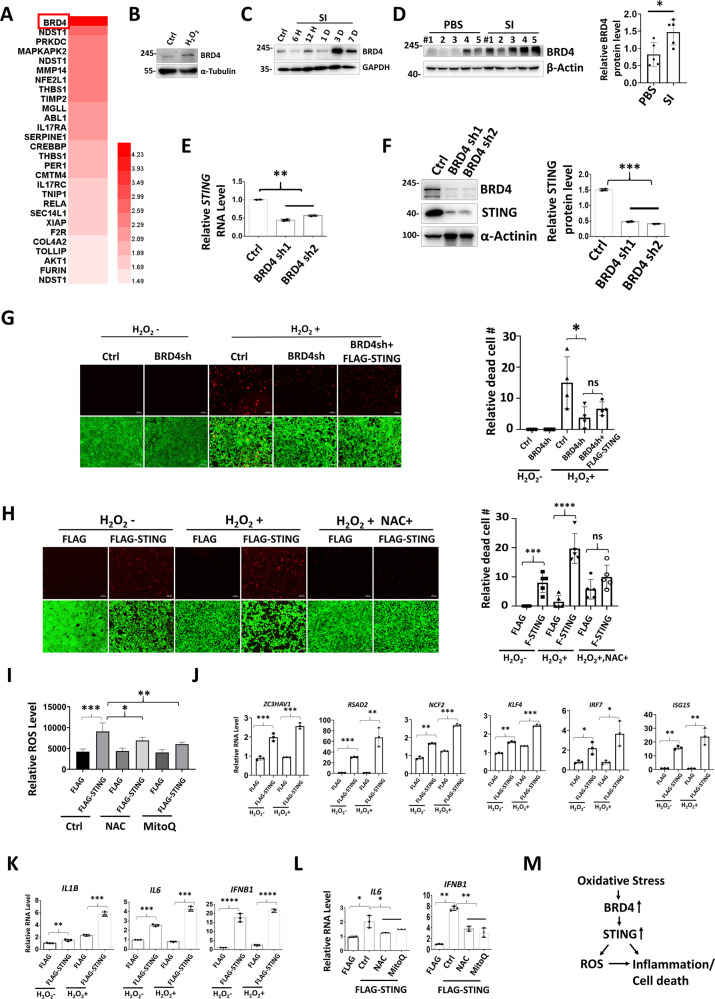
OS-induced BRD4 promotes STING expression and inflammation. **A** Heat map depicting upregulated genes involved in inflammatory response in OS-exposed ARPE cells (*p* < 0.05, with 1.5-fold change) [40]. **B**–**D** WB analysis showing induction of BRD4 upon OS. **B** 661W cells were treated with or without 600 μM H_2_O_2_ for 2 h and recover for 1 day before analysis. Mouse retinas were collected at the indicated time points (**C**) or 3 days (**D**) after SI injection for WB. qRT-PCR (**E**) or WB (**F**) analysis in ARPE cells. **G**, **H** Live/dead cell viability assay shows dead (red) and live (green) ARPE cells. For H_2_O_2_ treatment, 1.8 mM used in **G** and 0.6 mM used in **H**. **I** Relative ROS levels in ARPE cells. ROS levels were normalized by reads from cell counting kit-8 assay. **J**–**L** qRT-PCR showing gene expression in ARPE cells. After transfection, cells were treated with or without H_2_O_2_ (0.6 mM, 2 h) (**K**), or NAC (1 mM) or MitoQ (1 μM) for 6 h before analysis. **M** Schematic diagram summarizes OS-induced BRD4 activates STING and inflammation.

Analysis of our previous transcriptome dataset revealed BRD4 as the top upregulated inflammatory gene upon OS exposure in RPE cells [40] (Fig. 6A). Increased BRD4 was further confirmed in H_2_O_2_-treated 661 cells and in SI-injected mouse retinas (Fig. 6B–D). Moreover, BRD4 knockdown decreases STING expression (Fig. 6E, F), and prevented OS-induced cell death, which was reversed by STING overexpression (Fig. 6G). Addition of NAC suppresses cell death triggered by STING, suggesting involvement of ROS (Fig. 6H). Indeed, in line with recent report that STING loss reduces ROS and ROS-related genes [41], overexpression of STING promotes ROS generation and genes involved in ROS homeostasis (Fig. 6I, J). Finally, STING induced inflammatory factor expression, whereas treatment of NAC or MitoQ represses STING-induced IL6 and IFNβ (Fig. 6K, L). Together, we propose that OS-induced BRD4 activates STING and hence ROS production and inflammation (Fig. 6M).

### JQ1 inhibits SI-induced cGAS-STING activation, retina inflammation and degeneration

**Fig. 7:**
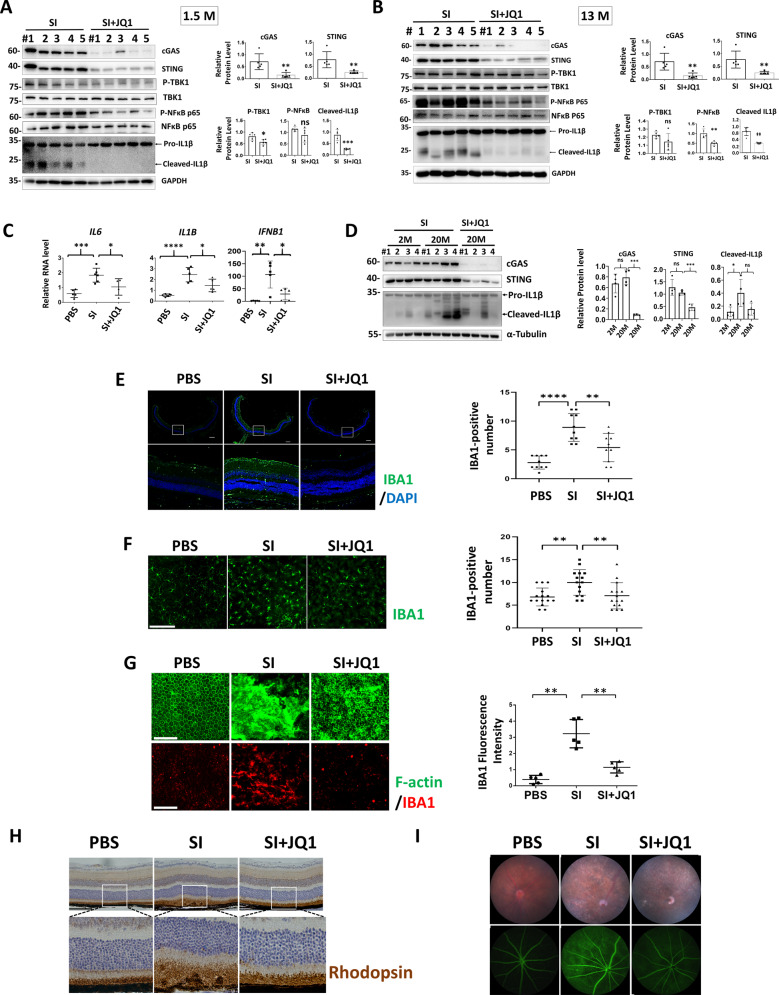
JQ1 inhibits cGAS-STING activation and retina degeneration after SI injury. WB (**A**, **B**, **D**) and qRT-PCR (**C**) analysis of indicated protein or gene expression. IF showing IBA1-postive cells in retina cryosections (**E**) and retina flat mounts (**F**). Right panels: quantification results of IBA1-positive cells in 10 (**E**) or 15 (**F**) randomly selected regions. Scale bar: 200 μM, *n* = 3. **G** RPE flat mounts were stained with IBA1 and FITC Phalloidin to label F-actin. Scale bar: 100 μM. Right panels: quantification results of IBA1-positive cells in five randomly selected regions. **H** IHC analysis of the mouse retina. *n* = 4. Scale bar: 100 μM. **I** Fundus photography (upper panels) and fluorescein angiography (lower panels) analysis. *n* = 4.

Treatment of JQ1 in young and middle-aged mouse reduced cGAS-STING activation after SI injection (Fig. 7A–C). Importantly, the inhibitory effects of JQ1 on cGAS, STING and IL1β were also detected in aged mouse retinas, indicating JQ1 also functions in retinal inflammation of old mice, which is more relevant to AMD pathogenesis (Fig. 7D). Next we determined the effect of JQ1 on retina protection upon SI injury. We found JQ1 repressed immune cell accumulation in retina and RPE (Fig. 7E–G), prevented RPE and retina degeneration (Fig. 7G, H), and improves retinal integrity (Fig. 7I) after SI injection. Importantly, JQ1 alone exhibited no detectable effects on retina morphology or retinal cell viability (Fig. S3). Together, our results indicate that JQ1 suppressed cGAS-STING activation and ameliorates retina inflammation and degeneration upon oxidative injury.

### JQ1 suppresses cGAS-STING pathway by promoting dsDNA clearance

**Fig. 8:**
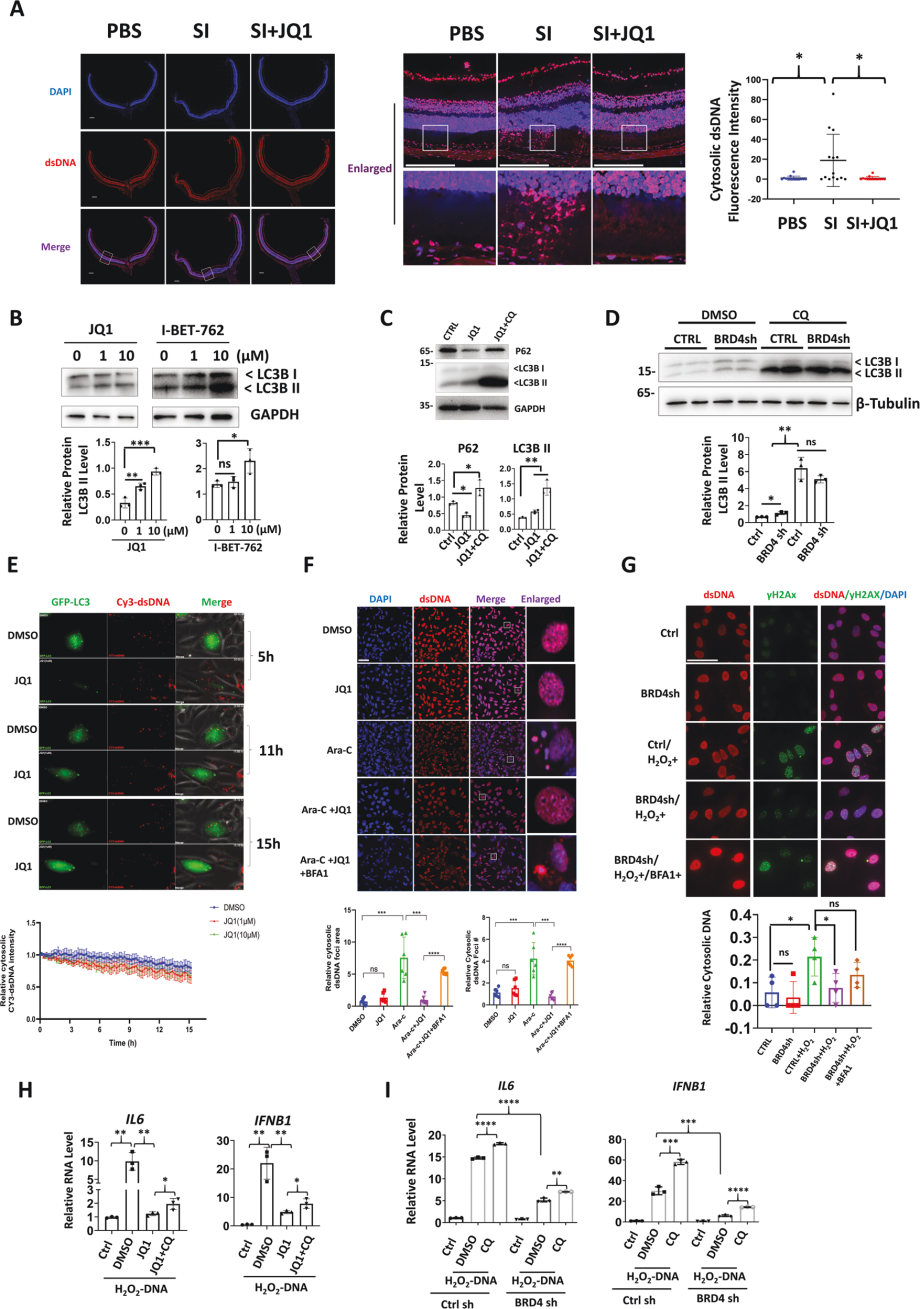
BRD4 inhibition promotes autophagy-dependent clearance of cytosolic DNA. **A** IF analysis showing cytosolic dsDNA in retina photoreceptors. Scale bar: 200 μM. WB analysis showing indicated proteins in 661W cells (**B**) or ARPE cells (**C**, **D**). **E** Live cell imaging showing JQ1 promoted exogenous cytosolic dsDNA clearance. Cy3- DNA was transfected into ARPE LC3-GFP cells and live cell imaging was performed 6 h after transfection with or without the addition of JQ1 (1 or 10 μM). The still frames were indicated at the indicated time points. Lower panel: mean fluorescence intensity of Cy3-dsDNA calculated from live cell images using ImageJ from 20 different fields. **F** IF for dsDNA in 661W cells. Scale bar: 50 μM. Lower panels: the relative cytosolic dsDNA foci number and area in the intact nuclei were calculated from six randomly selected fields. **G** IF analysis showing dsDNA and DNA damage. Scale bar: 20 μM. Lower panel: relative cytosolic DNA number after normalized to nuclei numbers. For each group, 30–50 cells were counted. **H**, **I** qRT-PCR analysis showing indicated gene expression in ARPE cells. JQ1 (10 μM), and or CQ (10 μM) were added 6 h after transfection.

IF analysis revealed that JQ1 inhibited SI-induced cytosolic leakage of dsDNA in photoreceptors (Fig. 8A). Interestingly, BRD4 has been recently reported as a transcription repressor for autophagy genes [22]. We thus hypothesize that JQ1 may also promote cytosolic DNA autophagy. Therefore, we first confirmed that JQ1 or BRD4 knockdown increased the levels of the lapidated form of LC3 (LC3II), which was more evident when the autophagic flux was blocked by chloroquine treatment (Fig. 8B–D). Next, we transfected Cy3-labeled dsDNA into RPE cell ARPE stably expressing GFP-LC3. Live cell images showed that Cy3-dsDNA was enclosed by the LC puncta and addition of JQ1 further accelerated LC3 puncta formation and DNA degradation (Fig. 8E and Supplementary Video 1). To further determine whether BRD4 inhibition enhanced clearance of endogenous cytosolic DNA, cytosolic dsDNA was induced by Ara-C. IF analysis indicates evident cytosolic dsDNA after Ara-C treatment, whereas JQ1 treatment led to the largely disappearance of cytosolic DNA, an effect that was blocked by the autophagy inhibitor Bafilomycin A1 (BafA1) (Fig. 8F). Similarly, BRD4 knockdown reduced cytosolic DNA leakage after H_2_O_2_ exposure, which was reversed by BafA1 treatment (Fig. 8G). Finally, we determined the effect of BRD4 inhibition on cytosolic DNA-induced inflammation. After transfection of H_2_O_2_-treated genomic DNA, JQ1 or BRD4 depletion profoundly reduced IL6 and IFNβ expression, whereas autophagy inhibitor CQ partially reversed such repression (Fig. 8H, I). Together, our data showed that BRD4 inhibition reduced cytosolic dsDNA accumulation and consequent inflammation in an autophagy-dependent manner.

## Discussion

Here, we have showed cytosolic DNA leakage in photoreceptor and activation of the cGAS-STING pathway in OS-induced retina degeneration. BRD4 was increased in injured retina and BRD4 inhibition ameliorates retina degeneration and inflammation, inhibits cGAS-STING activation, and promotes cytosolic DNA clearance.

GA is the major cause of blindness that currently lacks treatment. Elevated cGAS levels have been demonstrated in the RPE of GA patients, and causally related to RPE degeneration in a GA-like mouse model [16]. Here, we used SI, a stable oxidizing agent, to induce primary RPE injury followed by ensuing photoreceptor loss. This mouse model has been shown to mimic several clinical and histologic features of GA in AMD [26, 30]. After RPE injury, most cytosolic dsDNA and STING signals were observed in the inner and/or outer segments of photoreceptors. This result is reasonable considering rod and cone are the most prevalent cells in retina and make direct contact with the RPE. In degenerative RPE, cytosolic DNA is derived from mitochondria [16], whereas our results indicated that cytosolic DNA in damaged retina compromising both nuclear and mitochondrial DNA. Whether leakage of mitochondrial or nuclear DNA is an insult- or tissue-dependent response remains elusive.

Our analysis showed *cGAS* and *STING* genes are more accessible in the macular retinas of GA patients though global chromatin accessibility are decreased here, and *STING* is selectively increased in the macular retinas of dry AMD. Currently, directly correlation between chromatin opening and *STING* transcription activation in dry AMD is lacking, but epigenetic mechanism likely controls STING expression hence AMD pathogenies. Interestingly, an independent drug screening recently revealed BRD4 inhibitor I-BET prevented SI-induced RPE cell death in vitro [42], further highlighting beneficial effects of BRD4 inhibitor in GA. We also showed BRD4 inhibition suppressed stress-induced cytosolic DNA accumulation. BRD4 inhibition promoted autophagy of protein aggregates, but have no effect on autophagic removal of mitochondria or bacteria [22]. Here our results expand BRD4 inhibition in cytosolic DNA autophagy. More importantly, BRD4 inhibition potently repressed inflammation triggered by cytosolic DNA from H_2_O_2_-treated cells. Oxidized DNA are resistant to exonuclease degradation in the cytosol, hence potentiates enhanced immune recognition and inflammation as compared to unmodified DNA [17]. Given activation of ROS pathway in dry AMD and the prominent cytosolic DNA leakage after SI injury, clearance of oxidized DNA in cytosol may be an intriguing strategy for AMD treatment.

In summary, this study uncovered that cytosolic release of DNA and activation of cGAS-STING play key roles in retinal inflammation and degeneration. We propose that targeting cGAS-STING with BRD4 inhibition may provide new therapeutic approach for dry AMD and for other inflammatory injuries driven by cGAS-STING dysfunction.

## Materials and methods

### Animals

C57BL/6J mice 6–8-week-,13-month-old or 19–21-month-old were used in this study. The eye morphology was first confirmed normal under the light microscope or with fundus photography. Mice were housed in standard cages in a specific pathogen-free facility on a 12-h light/dark cycle with ad libitum access to food and water. For each experiment, mice of the similar age were randomly allocated, except for the age-related studies, in which the mice were divided into different groups according to the age. Male and female mice were randomly used for sex is generally irrelevant to eye morphology. For SI injection, a sterile 0.5% SI solution was freshly prepared, diluted in PBS and IP injected into mice as previously described (35 mg/kg) [25]. Control mice were injected with the same volumes of PBS. At 2 h post SI injection, JQ1 (50 mg/kg) or vehicle (2% DMSO, 30% Polyoxy ethylene300, and 5% Tween80) was IP injected and the injection was performed daily for 3 days. For diABZI treatment, mice were injected with PBS or SI (35 mg/kg) and after 1 h, a single dose of diABZI (3 mg/kg) or vehicle was IP injected and analysis was then performed 3-day after injection. For C176 treatment, mice were IP injected with SI (35 mg/kg) and after 1 h, C176 (10 mg/kg) or vehicle was IP injected, and then injected daily for 3 days. For WB analysis, at least 5 eyes from individual mouse were used for each group. For HE, IHC or IF analysis, at least three eyes from individual mouse were studied. All experimental procedures involving animals were approved by Animal Use and Care Committee of Zhongshan Ophthalmic Center at the Sun Yat-Sen University, Guangzhou, China.

### Fundus photography and fluorescein angiography

Fundus images were obtained as previously described [40]. Briefly, fluorescein angiography was performed before or after SI injection using the Micron IV retinal imaging microscope (Phoenix Research Laboratories, Pleasanton, CA, USA). After anesthesia and dilation of the pupils, the mice were IP injected with 2% fluorescein sodium solution (Alcon laboratories, TX, USA) (5 μl/g), and fluorescein angiographic images were recorded immediately.

### Histology, immunohistochemistry and immunofluorescence

For immunohistochemistry (IHC), the eyes were fixed in FAS eye fixation solution (Servicebio# G1109), dehydrated using an increasing ethanol gradient and embedded in paraffin as previously described [40]. Three sections (thickness: 8 μm) through the optic disk of each eye were prepared. The antigen was retrieved by incubation at 95 °C in 10 mM sodium citrate buffer for 30 min, after which the slides were immunostained with primary antibodies or normal rabbit IgG at 4 °C overnight. The following IHC was conducted according to the manufacturer’s protocol (GTVision TMIII #GK500705). After development, the slides were counterstained with or without hematoxylin and observed under a ZEISS Axio Observer 3 microscope. For mouse RPE IF, the procedure was performed as described previously [40]. The RPE flat mounts were incubated with primary antibodies overnight at 4 °C, followed by a 2-h incubation with the secondary antibody. F-actin was labeled with fluorescein isothiocyanate phalloidin (YEASEN # 40735ES75). Images were captured with a Tissue Fax confocal microscope. The antibodies and the dilutions are listed in Table 1.

**Table 1:** Key reagents and resources used in this study.

Reagent or resource	Source	Identifier
Antibodies		
STING (D1V5L) Rabbit mAb (Rodent Preferred)	Cell Signaling Technology	cat#50494
Phospho-STING (Ser365) (D8F4W) Rabbit mAb	Cell Signaling Technology	cat#72971
cGAS (D3O8O) Rabbit mAb (Mouse Specific)	Cell Signaling Technology	cat#31659
TBK1/NAK (D1B4) Rabbit mAb	Cell Signaling Technology	cat#3504
Phospho-TBK1/NAK (Ser172) (D52C2) XP® Rabbit mAb	Cell Signaling Technology	cat#5483
IRF-3 (D83B9) Rabbit mAb	Cell Signaling Technology	cat#4302
Phospho-IRF-3 (Ser396) (D6O1M) Rabbit mAb	Cell Signaling Technology	cat#29047
Phospho-NF-κB p65 (Ser536) (93H1) Rabbit mAb	Cell Signaling Technology	cat#3033
NF-κB p65 (D14E12) XP® Rabbit mAb	Cell Signaling Technology	cat#8242
IL-1β (3A6) Mouse mAb	Cell Signaling Technology	cat#12242
Phospho-Histone H2A.X (Ser139) (20E3) Rabbit mAb	Cell Signaling Technology	cat#9718
GAPDH (D16H11) XP® Rabbit mAb	Cell Signaling Technology	cat#5174
Acetyl-Histone H3 (Lys9) (C5B11) Rabbit mAb	Cell Signaling Technology	cat#9649
Histone H3 (D1H2) XP® Rabbit mAb	Cell Signaling Technology	cat#4499
Phospho-IRF-7 (Ser437/438) (D6M2I) Rabbit mAb (Mouse Specific)	Cell Signaling Technology	cat#24129
Anti-rabbit IgG (H+L), F(ab')2 Fragment (Alexa Fluor® 488 Conjugate)	Cell Signaling Technology	cat# 4412
Anti-mouse IgG (H+L), F(ab')2 Fragment (Alexa Fluor® 594 Conjugate)	Cell Signaling Technology	cat# 8890
Anti-rabbit IgG (H+L), F(ab')2 Fragment (Alexa Fluor® 594 Conjugate)	Cell Signaling Technology	cat# 8889
Rabbit monoclonal [EPR16588] to IBA1	Abacm	cat#ab178846
Rabbit polyclonal to Histone H3 (tri methyl K9)	Abacm	cat#ab8898
Rabbit polyclonal to RNA polymerase II CTD repeat YSPTSPS (phospho S2)	Abacm	cat#ab5095
Rabbit polyclonalto Brd4	Abacm	cat# ab84776
Rabbit polyclonal to LC3B	Abacm	cat# ab48394
Rabbit polyclonal to SQSTM1 / p62	Abacm	cat#ab155686
p-Histone H2A.X (Ser 139) anti-mouse	Santa Cruz Biotechnology	cat#sc-517348
dsDNA Marker (HYB331-01)	Santa Cruz Biotechnology	cat#sc-58749
Antibody anti-rabbit IRF-7 antibody(F-1)	Santa Cruz Biotechnology	cat#sc-74471
8-OHdG antibody (E-8)	Santa Cruz Biotechnology	cat# sc-393871
TMEM173/STING Antibody	Proteintech	cat#66680-1
GAPDH Mouse Monoclonal Antibody	Proteintech	cat#60004-1
beta Tubulin Mouse Monoclonal Antibody	Proteintech	cat#66240-1
TFAM Rabbit Polyclonal Antibody	Proteintech	cat# 22586-1
Alpha Actinin Polyclonal antibody	Proteintech	cat# 11313-2
Polyclonal Rabbit Anti- Glial Fibrillary Acidic Protein (GFAP)	Dako	cat#Z0334
Rhodopsin	ShuYi Chen Lab	cat#Mm53356
Phospho-Brd4(Ser492/Ser494)	Merck Millipore	cat# ABE1453
Rabbit anti-53BP1 Antibody	Bethyl	cat#A300-272A
FITC Phalloidin	Yeasen	cat# 40735ES75
Recoverin Antibody anti-rabbit	EMD Millipore	cat#AB5585
Chemicals, peptides, and recombinant proteins		
diABZI STING agonist (compound 3) (diABZI STING agonist (compound 3)	Selleck	cat#S8796
JQ1	Selleck	cat#S7110
Birabresib (OTX015)	Selleck	cat#S7360
Molibresib (I-BET-762)	Selleck	cat#S7189
PFI-2 HCl	Selleck	cat#S7294
MS436	Selleck	cat#S7294
Cerdulatinib (PRT062070)	Selleck	cat#S7634
Remodelin	Selleck	cat#S7641
Bafilomycin A1(BafA1)	Selleck	cat#S1413
Mitoquinone	Selleck	cat#S8978
Acetylcysteine (N-acetylcysteine)	Selleck	cat#S1623
Chloroquine	Selleck	cat#S6999
PEG300	Selleck	cat# S6704
cGAMP	Macklin	cat#G877072
DMSO	MP Biomedicals	cat#196055
Sodium iodate	Sigma-Aldrich	cat#S4007
Cy3-X-dUTP	ABP Biosciences	cat#C419
Hieff TransTM Liposomal transfection reagent	Yeasen	cat#40802ES03
Epigenetics Compound Library	Selleck	cat#L1900-Z308784
Experimental models: cell lines		
ARPE-19	ATAC	
661W	Huangxuan Shen Lab	N/A
human primary RPE cells	This study	N/A
Experimental Models: Organisms/Strains		
Mouse: C57BL/6: C57BL/6J	Sun Yat-Sen University	C57BL/6J
	Laboratory Animal Center	
Deposited data		
ATAC-seq Data	Gene Expression Omnibus	GSE99287
RNA Microarray Data	Gene Expression Omnibus	GSE29801
ChIP-seq Data	Gene Expression Omnibus	GSE21910
Software and algorithms		
GraphPad Prism 7.0	GraphPad software	https://www.graphpad.com/
ImageJ 1.46/ Fiji	NIH	https://imagej.nih.gov/ij/
IGV_2.8.2	Integrative Genomics Viewer	www.igv.org/
SRA Tools	NCBI	https://github.com/ncbi/sra-tools
Trim Galore	Babraham Institute	http://www.bioinformatics.babraham.ac.uk/projects/trim_galore/
SAMTools	Li et al. [46]	http://www.htslib.org/
Sambamba	Tarasov et al. [47]	https://github.com/biod/sambamba
MACS2	N/A	https://github.com/macs3-project/MACS
deepTools	Ramírez et al. [48]	https://deeptools.readthedocs.io/en/develop/index.html

### Mouse retina protein extraction and WB analysis

The retinas were dissected in PBS and suspended in 200 μl of radioimmunoprecipitation assay buffer (per retina) containing proteinase inhibitor cocktail and protein phosphatase inhibitor. The total proteins were extracted by sonication using a SCIENTZ-IID ultrasonic homogenizer (Amplitude: 60%, 1 s on and 1 s off for 1 min in total). For WB analysis, 25–50 μg of total protein was used. The phosphorylated proteins were normalized to their total proteins. The antibodies and the dilutions are listed in Table 1. All original WB results are shown in Supplementary Fig. S4.

### Nucleosome occupancy analysis

Nucleosome occupancy analysis was conducted as previously described with modification [43]. Briefly, 661W cells were harvested in ice-cold PBS and the nuclei were extracted by incubating with cell lysis buffer (10 mM Tris-HCl pH8.0, 10 mM MgCl_2_, 1 mM DTT and 0.5% NP-40) for 5 min on ice. After centrifugation, the pellets were suspended in digestion buffer (15 mM Tris-HCl pH 7.4, 60 mM KCl, 15 mM NaCl, 0.25 M sucrose, 1 mM CaCl_2_ and 0.5 mM DTT) and digested with micrococcal nuclease (100U/100 μl) for 15 min. The digestion was terminated by the addition of 20 μl stop buffer (100 mM EDTA, 10 mM EGTA, pH 7.5). The digested chromosomes were further treated with RNaseA and Proteinase K and extracted with a Universal DNA purification kit (Tiangen, #DP214). The DNA was then subjected to real-time PCR and the primers used are listed in Table 2. To account for differences in primer efficiency and DNA concentration, the data were normalized to genomic DNA of each sample and are expressed relative to the control treatment.

**Table 2:** Primers used for qRT-PCR, qCHIP, MNase assay and mitochondria and nuclear DNA detection.

Primer for specific gene	Primer direction	Primer sequence
Human IL1β	F	CTCGCCAGTGAAATGATGGCT
Human IL1β	R	GTCGGAGATTCGTAGCTGGAT
Human IL6	F	GTGAAAGCAGCAAAGAGGC
Human IL6	R	TTTCACCAGGCAAGTCTCC
Human IL8	F	TTGGCAGCCTTCCTGATTT
Human IL8	R	AACTTCTCCACAACCCTCTGC
Human IFNB	F	GCTCTCCTGTTGTGCTTCTCC
Human IFNB	R	CCTCCTTCTGGAACTGCTGC
Human cGAS	F	ACGTGCTGTGAAAACAAAGAAG
Human cGAS	R	GTCCCACTGACTGTCTTGAGG
Human STING	F	ATATCTGCGGCTGATCCTGC
Human STING	R	GGTCTGCTGGGGCAGTTTAT
Human GAPDH	F	GAAGGTGAAGGTCGGAGT
Human GAPDH	R	GAAGATGGTGATGGGATTTC
Mouse IL1β	F	TGCAGACTCAAACTCC
Mouse IL1β	R	TGAAAGACGGCACACC
Mouse IL6	F	GTTCTCTGGGAAATCGTGG
Mouse IL6	R	CTGCAAGTGCATCATCGTT
Mouse IFNB	F	CTCCAGCTCCAAGAAAGGAC
Mouse IFNB	R	TGGCAAAGGCAGTGTAACTC
Mouse cGAS	F	GGAAGGAACCGGACAAGCTA
Mouse cGAS	R	AACTCCGACTCCCGTTTCTG
Mouse STING	F	GGAACACCGGTCTAGGAAGC
Mouse STING	R	TGGATCCTTTGCCACCCAAA
Mouse mitochondria	F	CTAGAAACCCCGAAACCAAA
Mouse mitochondria	R	CCAGCTATCACCAAGCTCGT
Mouse B2M	F	ATGGGAAGCCGAACATACTG
Mouse B2M	R	CAGTCTCAGTGGGGGTGAAT
Mouse ACTIN	F	CCTAAGGCCAACCGTGAAAA
Mouse ACTIN	R	CAGAGGCATACAGGGACAGC
Mouse GAPDH promoter	F	CCACTTGTGGCAAGAGGCTA
Mouse GAPDH promoter	R	GTGGAGAGTTGGGACGTGAG
Mouse cGAS (MNase)#1	F	AGGAGCAAAATTCACTGCGA
Mouse cGAS (MNase)#1	R	CCCACAGGTGATGCTAAGAG
Mouse cGAS (MNase)#2	F	TGGAATAGGCATGAGCATCG
Mouse cGAS (MNase)#2	R	GTCGCAGTGAATTTTGCTCC
Mouse cGAS (MNase)#3	F	TCGGTGTCTTTTTATTCAGGCT
Mouse cGAS (MNase)#3	R	TGCAATCCTGTGTGTCCCTT
Mouse cGAS (MNase)#4	F	TTGGCTGCTGAGATTCCGTA
Mouse cGAS (MNase)#4	R	GCAAAATGAGTTCCGCCAAG
Mouse cGAS (MNase)#5	F	GGTTTACAGTGAGTCCCAGGAC
Mouse cGAS (MNase)#5	R	TGGCTAGATTTGCCGCCTAC
Mouse STING (MNase)#1	F	CGTTTAAAGAGCCAGGCAGTG
Mouse STING (MNase)#1	R	TGGATTGTGGTCTGCACGTT
Mouse STING (MNase)#2	F	CAGATGGCTAGCAGGGAAGAG
Mouse STING (MNase)#2	R	GGAGGGCACCGGACAATTTAT
Mouse STING (MNase)#3	F	TTTCGGGGAAATAACCACGC
Mouse STING (MNase)#3	R	GGACCTGGACTTCCCTTCAT
Mouse STING (MNase)#4	F	GGCGTGGTTATTTCCCCGAA
Mouse STING (MNase)#4	R	GGGGAGGGGTTAGACAGGAG
Mouse STING (MNase)#5	F	GCTTTGGCAGGAAACACCAAA
Mouse STING (MNase)#5	R	AACTGCAACTCAGCTCGCTT
Mouse STING (ChIP)#1	F	GCCAGATGGCTAGCAGGGAA
Mouse STING (ChIP)#1	R	TGGGTATCAGGGATCCAACAC
Mouse STING (ChIP)#2	F	TTTCGGGGAAATAACCACGC
Mouse STING (ChIP)#2	R	GGACCTGGACTTCCCTTCAT
Mouse STING (ChIP)#3	F	GGCGTGGTTATTTCCCCGAA
Mouse STING (ChIP)#3	R	GGGGAGGGGTTAGACAGGAG
Mouse STING (ChIP)#4	F	GCTTTGGCAGGAAACACCAAA
Mouse STING (ChIP)#4	R	AACTGCAACTCAGCTCGCTT
Human ISG15	F	AATGCGACGAACCTC
Human ISG15	R	GCTCACTTGCTGCTT
Human NCF2	F	ACTGCCTGACTCTGTGGT
Human NCF2	R	ACTTGGCTGCCTTTCTTA
Human IRF7	F	TACCTGTCACCCTCCCC
Human IRF7	R	GTCCCACCACCTTCTGC
Human KLF4	F	CTGAGCGGGCGAATTTCCATC
Human KLF4	R	CGGGCTGCGGCAAAACCTACA
Human ZC3HAV1	F	GATGGACAGAAAGGTG
Human ZC3HAV1	R	CGATGTGAAGAAGGAG
Human RSAD2	F	CGGAACAGATCAAAGCACT
Human RSAD2	R	TTAGATTCAGGCACCAAGC
Human ACTIN	F	TCACCAACTGGGACGACAT
Human ACTIN	R	ATCTGGGTCATCTTCTCGC
Human TFAM	F	CGCTCCCCCTTCAGTTTTGT
Human TFAM	R	CCAACGCTGGGCAATTCTTC
Human BRD4	F	TGCTGACGTCCGATTGATGT
Human BRD4	R	TCGAACACATCCTGGAGCTTG
Human Trp53bp1	F	CAAAGAATTCTGGACTGGCAACCC
Human Trp53bp1	R	TCCAGGAAGTTCTGCTGTTGGTC
Human BRD4 (shRNA)#1	F	CCGGCAGTGACAGTTCGACTGATGACTCGAGTCATCAGTCGAACTGTCACTGTTTTTG
Human BRD4 (shRNA)#1	R	AATTCAAAAACAGTGACAGTTCGACTGATGACTCGAGTCATCAGTCGAACTGTCACTG
Human BRD4 (shRNA)#2	F	CCGGCCTGGAGATGACATAGTCTTACTCGAGTAAGACTATGTCATCTCCAGGTTTTTG
Human BRD4 (shRNA)#2	R	AATTCAAAAACCTGGAGATGACATAGTCTTACTCGAGTAAGACTATGTCATCTCCAGG
Human Trp53bp1 (shRNA)#1	F	CCGGCCCTTGTTCAGGACAGTCTTTCTCGAGAAAGACTGTCCTGAACAAGGGTTTTTG
Human Trp53bp1 (shRNA)#1	R	AATTCAAAAACCCTTGTTCAGGACAGTCTTTCTCGAGAAAGACTGTCCTGAACAAGGG
Human Trp53bp1 (shRNA)#2	F	CCGGGATACTCCTTGCCTGATAATTCTCGAGAATTATCAGGCAAGGAGTATCTTTTTG
Human Trp53bp1 (shRNA)#2	R	AATTCAAAAAGATACTCCTTGCCTGATAATTCTCGAGAATTATCAGGCAAGGAGTATC
Human NC (shRNA)	F	CCGGAAGCTGGAGTACAACTACAACCTCGAGGTTGTAGTTGTACTCCAGCTTTTTTTG
Human NC (shRNA)	R	AATTCAAAAAAAGCTGGAGTACAACTACAACCTCGAGGTTGTAGTTGTACTCCAGCTT

### Drug screening

The screening process is shown in Fig. 4A. X-ray irradiation was performed using RS2000 irradiator (Rad Source, Technologies, Suwanee, GA, USA). The epigenetic drug library was purchased from Selleck (#L1900) and IF-based analysis was performed using the Operetta CLS high-content analysis system (PerkinElmer).

### Live cell imaging

DNA labeling was achieved by using PCR-based labeling procedure. Briefly, DNA template, primers and Green Taq Mix (Vazyme#P131) were incubated with 1 μl of Cy3-X-dUTP (1 mM) (ABP Biosciences #C419B), and the DNA was labeled using conventional PCR. The resulting DNA products were purified by using a Universal DNA purification kit (Tiangen, #DP214). For live cell imaging, ARPE-19 cells stably expressing GFP-LC3 were cultured in a 6-well plate. Cells were transfected with 1 μg/ml Cy3-labled DNA. At 6 h post transfection, cells were washed twice by pre-warmed PBS, and JQ1 (1 or 10 μΜ) was added into the growth medium, and live images were acquired by using Lioheart FX automated microscope (BioTek) and Cy3 fluorescence signals were quantified using ImageJ.

### DNA isolation and stimulation of cells in vitro

ARPE or 661 cells were treated with or without 1.2 mM of H_2_O_2_ for 2 h, recovered for 12 h before DNA extraction with a genomic extraction kit (TIANGEN, #DP304). ARPE or 661W cells were transfected with genomic DNA (2 μg/ml) using lipofectamine 2000. At 6 h post transfection, the medium was replaced with growth medium containing DMSO or JQ1 (10 μΜ) or CQ (1 μM) or a combination of JQ1 (10 μM) and CQ (1 μM). Cells were further cultured for 14–16 h before analysis.

### Intracellular ROS detection, CCK-8 analysis and cell viability assay

The intracellular ROS was determined by using a DCFDA/H2DCFDA-Cellular ROS Assay Kit (Abcam#113851) (2′,7′-dichlorofluorescin diacetate) according to the manufactory’s protocol. ARPE cells were transfected with indicated plasmid and then treated with DMSO or NAC (1 mM) or MitoQ (1 μM) for 6 h. The viable cell amount was determined using CCK-8 (Dojindo Molecular Technologies, #CK04). The resulting signal was recorded using Synergy H1 Hybrid Multi-Mode Microplate Reader (BioTek Instruments, USA). Cell viability was determined using LIVE/DEAD™ Viability/Cytotoxicity Kit (Invitrogen, #L3224) and cells were observed under a ZEISS Observer7 inverted microscope (Carl Zeiss). Cells were transfected with FLAG or FLAG-STING plasmids. After 24 h, cells were treated with or without H_2_O_2_ (1.8 mM: Fig. 6G, 0.6 mM: Fig. 6H.) for 2 h and then recovered for 12 h before analysis. For quantification, dead cells were normalized to the fluorescence intensity of live cells.

NAC (1 mM) was added 2 h before H_2_O_2_ treatment and then maintained throughout the experiment.

### ChIP assay

ChIP assay using mouse retinas was performed according to procedures described previously [44]. Briefly, three retinas were pooled together and homogenized in 250 μl of ice-cold PBS containing proteinase cocktails, then another 750 μl of PBS was added and cross-linking was performed with 1% formaldehyde (final concentration). Sonication was performed by using SCIENTZ ultrasonic homogenizer (Amplitude: 60%, 2 s on and 2 s off for 5 min in total) and 6 μg of chromatin were used for each immunoprecipitation. The antibodies and the dilutions are listed in Table 1, and the primers used are listed in Table 2.

### Reverse transcription-PCR (RT-PCR) and quantitative PCR (qPCR)

Both RT-PCR and qPCR were conducted as previously described [25]. Total RNA was extracted using an RNAprep Pure Kit (Tiangen #DP430) wherein the genomic DNA was removed by DNase I digestion. For cDNA synthesis, 1 μg of total RNA was used along with the RevertAid First Strand cDNA Synthesis Kit (Thermo #K1622) were used. The gene expression levels were analyzed using SuperReal PreMix Plus (SYBR Green) (Tiangen #FP205) and the LightCycler® 480 System (Roche). The assays were performed in triplicate and the Ct values were normalized to that of beta actin or GAPDH as indicated. The primers used are listed in Table 2.

### Cell culture and treatment

The cells used in this study were authenticated by STR profiling and have been tested for mycoplasma contamination. ARPE-19 or 661W cells were cultured in DMEM/F12 containing 10% fetal bovine serum and 1% penicillin-streptomycin. For Ara-C treatment, 661W cells were seeded on cover slides and treated with 10 μΜ of Ara-C (MedChemExpress, #HY-13605) for 12 h, and then the cells were either treated with or without JQ1 (1 μΜ) for 6 h or left untreated. cells were then fixed with 4% paraformaldehyde and subjected to IF analysis. To induce cytosolic DNA leakage, 661W cells were treated with or without 600 μM H_2_O_2_ for 2 h and then allowed to recover for 3 days before IF analysis. To determine the effect of BRD4 inhibitors on cGAS-STING transcription, 661W Cells were pretreated with 10 μM of JQ1, I-BET or OTX for 24 h, then subjected to IR (8 Gy) and harvested 24 h post-treatment. Alternatively, 661W cells were treated with Ara-C (10 μM) for 24 h and then BRD4 inhibitors (10 μM) were added for additional 48 h before analysis. To determine the autophagy of JQ on cytosolic DNA, 661W Cells were treated with DMSO, JQ1 (1 μM, 18 h) or Ara-C (10 μM, 18 h) as indicated. For Ara-C + JQ1 group, cells were pretreated with 10 μM of Ara-C, and then 1 μM of JQ1 was added in the presence of Ara-C for another 6 h.

### Separation of cytosolic and nuclear fractions

The procedure was conducted as previously described [45]. Briefly, for each sample, two mouse retinas were homogenized in 500 μl of ice-cold solution I (0.3 M sucrose, 2% Tween 20, 10 mM HEPES PH7.9, 10 mM KCl, 1.5 mM MgCl_2_, 0.1 mM EDTA) by pipetting. Then, the obtained suspension was gently added onto 500 μl of solution II (1.5 M sucrose, 10 mM HEPES Ph7.9, 10 mM KCl, 1.5 mM MgCl_2_, 0.1 mM EDTA). The nuclei and cytosol fractions were then separated by centrifugation (13,000 rpm, 4 °C, 10 min) and verified by WB analysis. The supernatant containing the cytosolic fractions was transferred to a new tube and the DNA were obtained by phenol chloroform extraction.

### Quantification of cytosolic DNA

Cytosolic DNA was quantified using a qPCR-based method. Briefly, a standard curve was created by serial dilution of mouse genomic DNA from 0.00325 to 10 ng. For qPCR analysis, cytosolic DNA (0.4 ng or 10 ng) was used, the obtained CP-values were plotted to the standard curve, and the relative concentration was calculated.

### ATAC-seq and GSEA analysis

ATAC-seq data ­­were accessed from NCBI’s Gene Expression Omnibus (GEO) under accession number GSE99287. SRA files were transformed to FASTQ format by SRA-Tools and adapters were removed by Trim Galore. 50 bp paired-end ATAC-Seq reads were aligned to the human reference genome (GRCh37/hg19) using Bowtie2 with default parameters. After filtering reads from mitochondrial DNA and the Y chromosome, we included properly paired reads with high mapping quality (MAPQ score >30, qualified reads) through SAMTools for further analysis. Duplicate reads were removed through Sambamba. ATAC-Seq peak regions of each sample were called using MACS2 with parameters --nomodel --shift -100 --extsize 200. To visualize chromatin accessibility changes between different groups, the BAM flies were merged by SAMTools, then normalized by deepTools with parameters bamCoverage --normalizeUsing CPM –exactScaling. For GSEA analysis, microarray data were accessed from NCBI’s GEO under accession number GSE29801 and analyzed by using the GSEA v4.1.0 software with the MSigDB c5.go.bp.v7.4.symbols.gmt. Gene set size filters (min = 15, max = 1000) resulted in filtering out 3417/7481 gene sets. Enriched gene sets were selected on the basis of statistical significance (false discovery rate FDR *q* value <0.25, and normalized *p* value <0.05).

### Statistical analysis

Results are expressed as mean ± SEM unless otherwise indicated. GraphPad Prism 8.0 software (GraphPad software, Inc., La Jolla, CA) was used for statistical analysis as described within Results. All tests are two-tailed, unpaired *t*-test unless otherwise indicated. *p < 0.05; **p < 0.01; ***p < 0.0001.

### Reporting summary

Further information on research design is available in the Nature Research Reporting Summary linked to this article.

## Supplementary information

Supplementary Figure 1

Supplementary Figure 2

Supplementary Figure 3

Supplementary Figure 4

Supplementary figure and table legends

Supplementary Video

Revised Bullet Points

Pre-Authorship form

Reporting Summary

## Data Availability

The datasets generated during and/or analyzed during the current study are available from the corresponding author on reasonable request.

## References

[1] Ambati J, Fowler BJ (2012). Mechanisms of age-related macular degeneration. Neuron..

[2] Holz FG, Strauss EC, Schmitz-Valckenberg S, van Lookeren Campagne M (2014). Geographic atrophy: clinical features and potential therapeutic approaches. Ophthalmology..

[3] Akhtar-Schafer I, Wang L, Krohne TU, Xu H, Langmann T (2018). Modulation of three key innate immune pathways for the most common retinal degenerative diseases. EMBO Mol Med.

[4] Boyer DS, Schmidt-Erfurth U, van Lookeren Campagne M, Henry EC, Brittain C (2017). The pathophysiology of geographic atrophy secondary to age-related macular degeneration and the complement pathway as a therapeutic target. Retina..

[5] Cabral de Guimaraes TA, Daich Varela M, Georgiou M, Michaelides M (2022). Treatments for dry age-related macular degeneration: therapeutic avenues, clinical trials and future directions. Br J Ophthalmol.

[6] Black JR, Clark SJ (2016). Age-related macular degeneration: genome-wide association studies to translation. Genet Med.

[7] Sun L, Wu J, Du F, Chen X, Chen ZJ (2013). Cyclic GMP-AMP synthase is a cytosolic DNA sensor that activates the type I interferon pathway. Science..

[8] Wu J, Sun L, Chen X, Du F, Shi H, Chen C (2013). Cyclic GMP-AMP is an endogenous second messenger in innate immune signaling by cytosolic DNA. Science..

[9] Chen Q, Sun L, Chen ZJ (2016). Regulation and function of the cGAS-STING pathway of cytosolic DNA sensing. Nat Immunol.

[10] Cai X, Chiu YH, Chen ZJ (2014). The cGAS-cGAMP-STING pathway of cytosolic DNA sensing and signaling. Mol Cell.

[11] Ishikawa H, Barber GN (2008). STING is an endoplasmic reticulum adaptor that facilitates innate immune signalling. Nature..

[12] Motwani M, Pesiridis S, Fitzgerald KA (2019). DNA sensing by the cGAS-STING pathway in health and disease. Nat Rev Genet.

[13] Maekawa H, Inoue T, Ouchi H, Jao TM, Inoue R, Nishi H (2019). Mitochondrial damage causes inflammation via cGAS-STING signaling in acute kidney injury. Cell Rep.

[14] Huang LS, Hong Z, Wu W, Xiong S, Zhong M, Gao X (2020). mtDNA activates cGAS signaling and suppresses the YAP-mediated endothelial cell proliferation program to promote inflammatory injury. Immunity..

[15] Li Q, Cao Y, Dang C, Han B, Han R, Ma H (2020). Inhibition of double-strand DNA-sensing cGAS ameliorates brain injury after ischemic stroke. EMBO Mol Med.

[16] Kerur N, Fukuda S, Banerjee D, Kim Y, Fu D, Apicella I (2018). cGAS drives noncanonical-inflammasome activation in age-related macular degeneration. Nat Med.

[17] Gehrke N, Mertens C, Zillinger T, Wenzel J, Bald T, Zahn S (2013). Oxidative damage of DNA confers resistance to cytosolic nuclease TREX1 degradation and potentiates STING-dependent immune sensing. Immunity..

[18] Beatty S, Koh H, Phil M, Henson D, Boulton M (2000). The role of oxidative stress in the pathogenesis of age-related macular degeneration. Surv Ophthalmol.

[19] Datta S, Cano M, Ebrahimi K, Wang L, Handa JT (2017). The impact of oxidative stress and inflammation on RPE degeneration in non-neovascular AMD. Prog Retin Eye Res.

[20] Belkina AC, Nikolajczyk BS, Denis GV (2013). BET protein function is required for inflammation: Brd2 genetic disruption and BET inhibitor JQ1 impair mouse macrophage inflammatory responses. J Immunol.

[21] Tasdemir N, Banito A, Roe JS, Alonso-Curbelo D, Camiolo M, Tschaharganeh DF (2016). BRD4 connects enhancer remodeling to senescence immune surveillance. Cancer Disco.

[22] Sakamaki JI, Wilkinson S, Hahn M, Tasdemir N, O’Prey J, Clark W (2017). Bromodomain protein BRD4 is a transcriptional repressor of autophagy and lysosomal function. Mol Cell.

[23] Zou M, Gong L, Ke Q, Qi R, Zhu X, Liu W (2022). Heterochromatin inhibits cGAS and STING during oxidative stress-induced retinal pigment epithelium and retina degeneration. Free Radic Biol Med.

[24] Newman AM, Gallo NB, Hancox LS, Miller NJ, Radeke CM, Maloney MA (2012). Systems-level analysis of age-related macular degeneration reveals global biomarkers and phenotype-specific functional networks. Genome Med.

[25] Gong L, Liu F, Xiong Z, Qi R, Luo Z, Gong X (2018). Heterochromatin protects retinal pigment epithelium cells from oxidative damage by silencing p53 target genes. Proc Natl Acad Sci USA.

[26] Moriguchi M, Nakamura S, Inoue Y, Nishinaka A, Nakamura M, Shimazawa M (2018). Irreversible photoreceptors and RPE cells damage by intravenous sodium iodate in mice is related to macrophage accumulation. Invest Ophthalmol Vis Sci.

[27] Enzmann V, Row BW, Yamauchi Y, Kheirandish L, Gozal D, Kaplan HJ (2006). Behavioral and anatomical abnormalities in a sodium iodate-induced model of retinal pigment epithelium degeneration. Exp Eye Res.

[28] Ma W, Zhang Y, Gao C, Fariss RN, Tam J, Wong WT (2017). Monocyte infiltration and proliferation reestablish myeloid cell homeostasis in the mouse retina following retinal pigment epithelial cell injury. Sci Rep.

[29] Sorsby A (1941). Experimental pigmentary degeneration of the retina by sodium iodate. Br J Ophthalmol.

[30] Hanus J, Anderson C, Wang S (2015). RPE necroptosis in response to oxidative stress and in AMD. Ageing Res Rev.

[31] Ramanjulu JM, Pesiridis GS, Yang J, Concha N, Singhaus R, Zhang SY (2018). Design of amidobenzimidazole STING receptor agonists with systemic activity. Nature..

[32] Haag SM, Gulen MF, Reymond L, Gibelin A, Abrami L, Decout A (2018). Targeting STING with covalent small-molecule inhibitors. Nature..

[33] Shen YJ, Le Bert N, Chitre AA, Koo CX, Nga XH, Ho SS (2015). Genome-derived cytosolic DNA mediates type I interferon-dependent rejection of B cell lymphoma cells. Cell Rep.

[34] Vizioli MG, Liu T, Miller KN, Robertson NA, Gilroy K, Lagnado AB (2020). Mitochondria-to-nucleus retrograde signaling drives formation of cytoplasmic chromatin and inflammation in senescence. Genes Dev.

[35] Hu M, Zhou M, Bao X, Pan D, Jiao M, Liu X (2021). ATM inhibition enhances cancer immunotherapy by promoting mtDNA leakage and cGAS/STING activation. J Clin Invest.

[36] Devaiah BN, Case-Borden C, Gegonne A, Hsu CH, Chen Q, Meerzaman D (2016). BRD4 is a histone acetyltransferase that evicts nucleosomes from chromatin. Nat Struct Mol Biol.

[37] Wang J, Zibetti C, Shang P, Sripathi SR, Zhang P, Cano M (2018). ATAC-Seq analysis reveals a widespread decrease of chromatin accessibility in age-related macular degeneration. Nat Commun.

[38] Jang MK, Mochizuki K, Zhou M, Jeong HS, Brady JN, Ozato K (2005). The bromodomain protein Brd4 is a positive regulatory component of P-TEFb and stimulates RNA polymerase II-dependent transcription. Mol Cell.

[39] Nicodeme E, Jeffrey KL, Schaefer U, Beinke S, Dewell S, Chung CW (2010). Suppression of inflammation by a synthetic histone mimic. Nature..

[40] Sun Q, Gong L, Qi R, Qing W, Zou M, Ke Q (2020). Oxidative stress-induced KLF4 activates inflammatory response through IL17RA and its downstream targets in retinal pigment epithelial cells. Free Radic Biol Med.

[41] Hayman TJ, Baro M, MacNeil T, Phoomak C, Aung TN, Cui W (2021). STING enhances cell death through regulation of reactive oxygen species and DNA damage. Nat Commun.

[42] Wang B, Wang L, Gu S, Yu Y, Huang H, Mo K (2020). D609 protects retinal pigmented epithelium as a potential therapy for age-related macular degeneration. Signal Transduct Target Ther.

[43] Gong L, Govan JM, Evans EB, Dai H, Wang E, Lee SW (2015). Nuclear PTEN tumor-suppressor functions through maintaining heterochromatin structure. Cell Cycle.

[44] Cotney JL, Noonan JP (2015). Chromatin immunoprecipitation with fixed animal tissues and preparation for high-throughput sequencing. Cold Spring Harb Protoc.

[45] Kawashima A, Tanigawa K, Akama T, Wu H, Sue M, Yoshihara A (2011). Fragments of genomic DNA released by injured cells activate innate immunity and suppress endocrine function in the thyroid. Endocrinology..

[46] Li H, Handsaker B, Wysoker A, Fennell T, Ruan J, Homer N (2009). The Sequence Alignment/Map format and SAMtools. Bioinformatics.

[47] Tarasov A, Vilella AJ, Cuppen E, Nijman IJ, Prins P (2015). Sambamba: fast processing of NGS alignment formats. Bioinformatics.

[48] Ramírez F, Ryan DP, Grüning B, Bhardwaj Vivek, Kilpert F, Richter AS (2016). deepTools2: a next generation web server for deep-sequencing data analysis. Nucleic Acids Res.

