# Supplementary figures and images for: Inhibition of cGAS-STING by JQ1 alleviates oxidative stress-induced retina inflammation and degeneration
Source: Cell Death Differ. 2022 Mar 28;29(9):1816–33. doi: 10.1038/s41418-022-00967-4 (PMC9433402; doi:10.1038/s41418-022-00967-4)

Fig. S1 IRF3 was not activated in mouse retina upon SI injection.

A

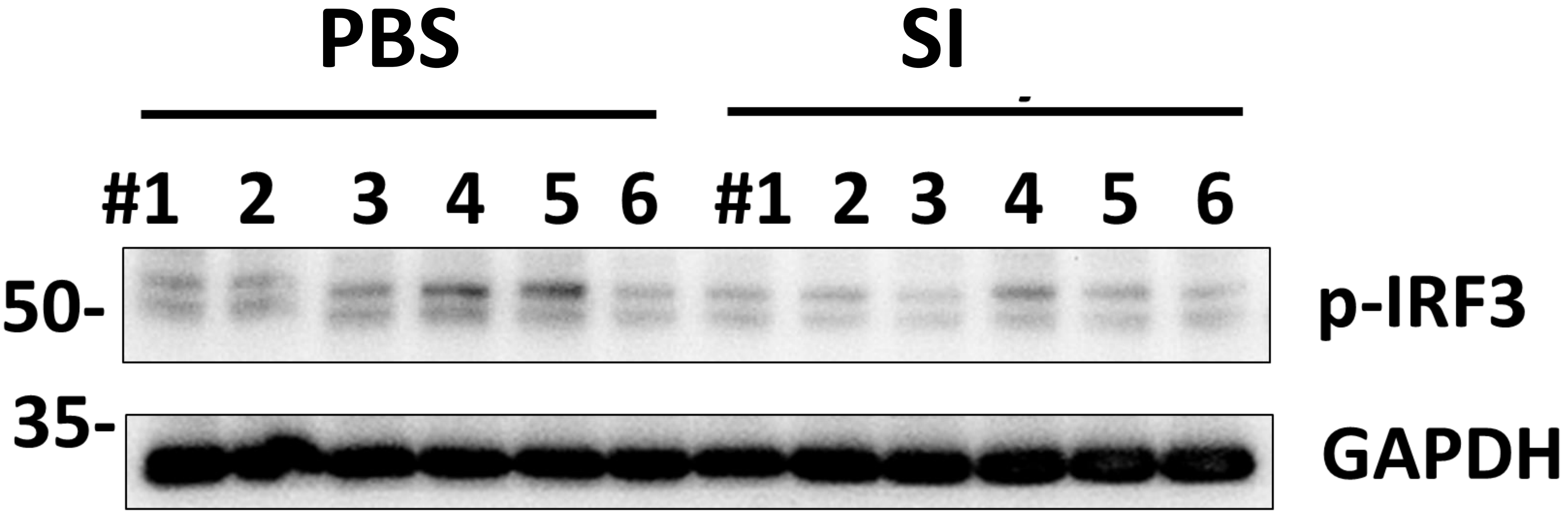

B

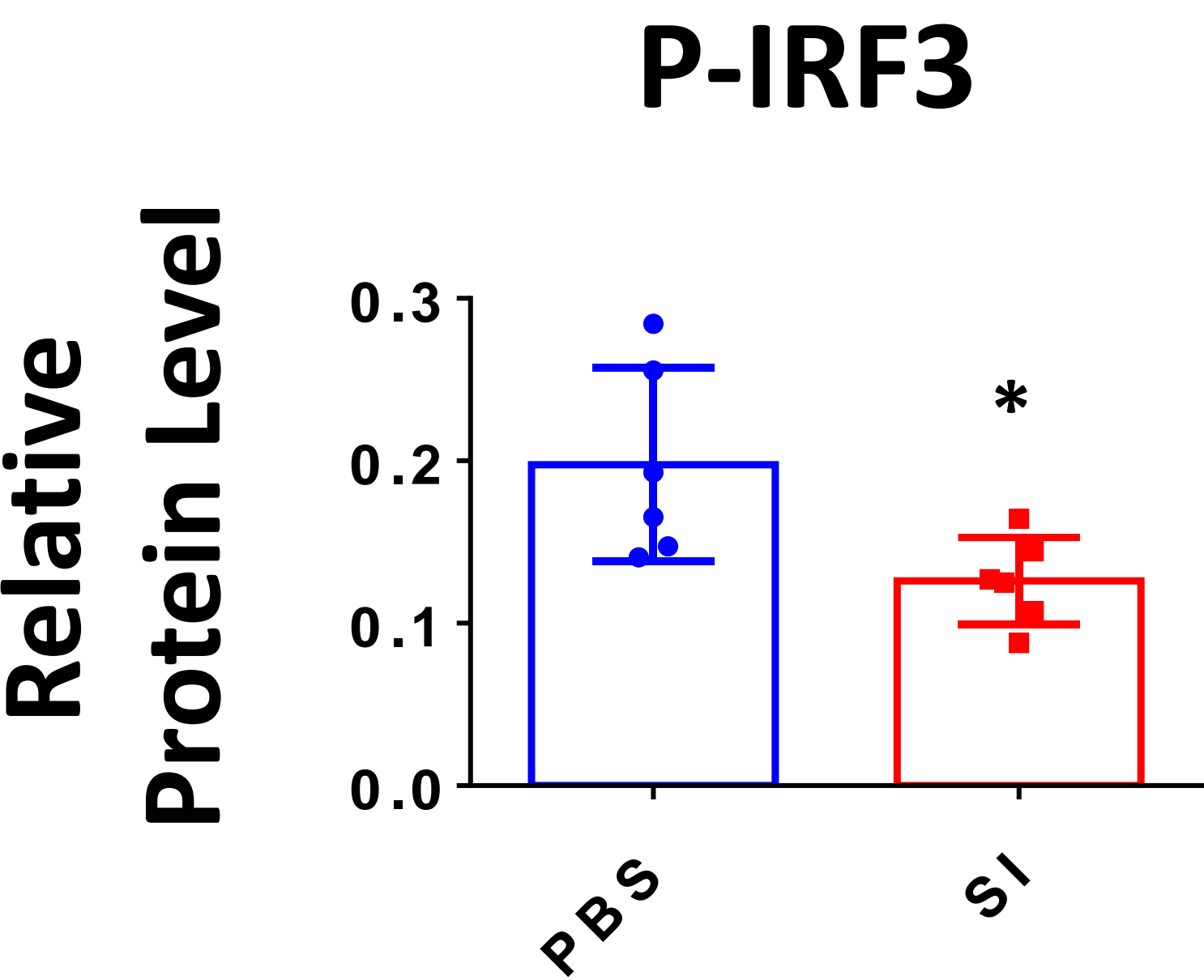

C

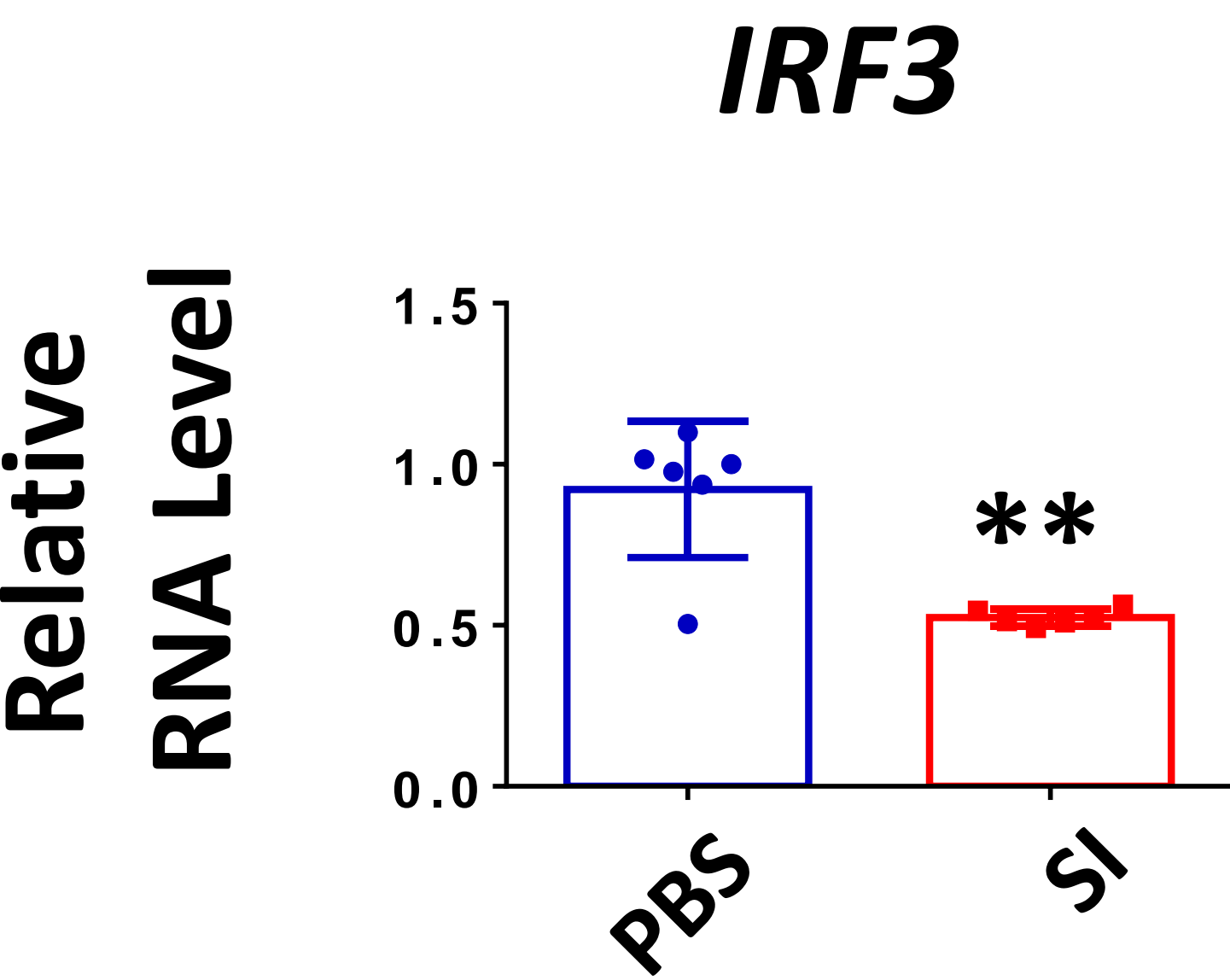

Supplement: Supplementary file 1 — Supplementary Figure 1 [file 41418_2022_967_MOESM1_ESM.pdf]

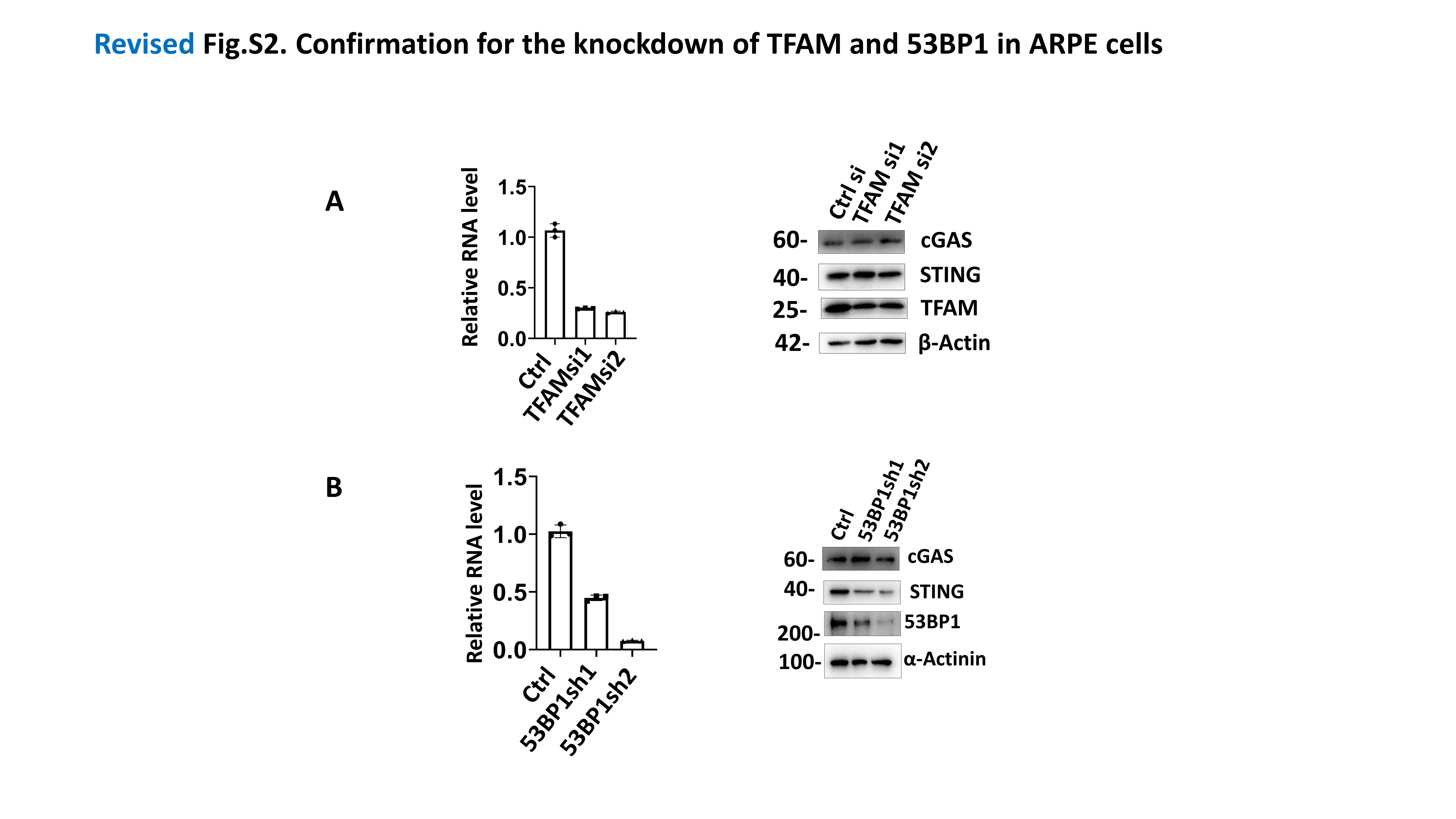

Supplement: Supplementary file 2 — Supplementary Figure 2 [file 41418_2022_967_MOESM2_ESM.tif]

Fig. S3 JQ1 does not alter eye morphology, retina structure or retinal cell viability

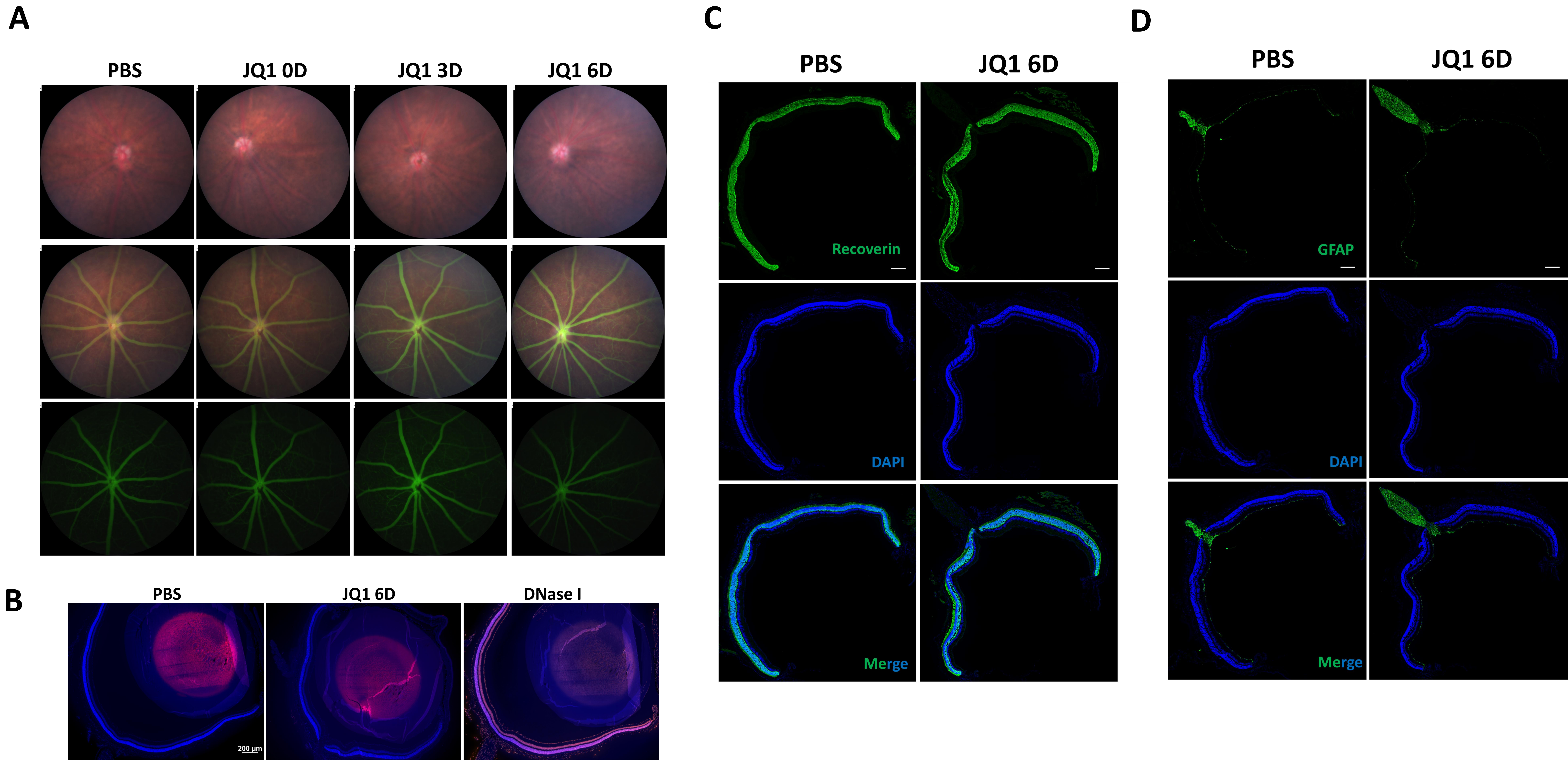

Supplement: Supplementary file 3 — Supplementary Figure 3 [file 41418_2022_967_MOESM3_ESM.pdf]
