# Supplementary material for: Inhibition of cGAS-STING by JQ1 alleviates oxidative stress-induced retina inflammation and degeneration
Source: Cell Death Differ. 2022 Mar 28;29(9):1816–33. doi: 10.1038/s41418-022-00967-4 (PMC9433402; doi:10.1038/s41418-022-00967-4)
Supplement: Supplementary file 4 — Supplementary Figure 4 [file 41418_2022_967_MOESM4_ESM.pdf]

Fig.1E

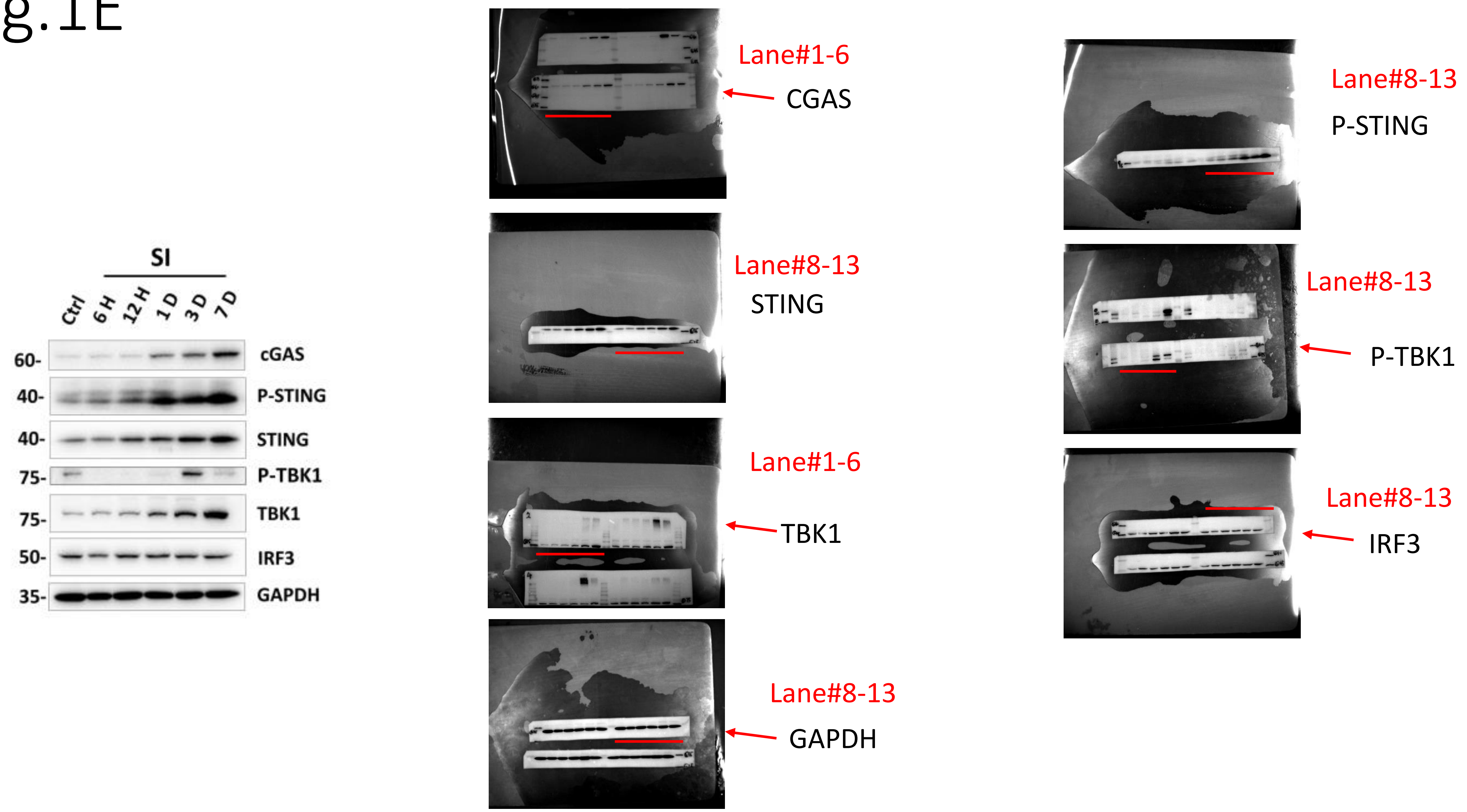
SI
Ctrl
6 H
12 H
1 D
3 D
7 D
60-
cGAS
40-
P-STING
40-
STING
75-
P-TBK1
75-
TBK1
50-
IRF3
35-
GAPDH
Lane#1-6
CGAS
Lane#8-13
STING
Lane#1-6
TBK1
Lane#8-13
GAPDH
Lane#8-13
P-STING
Lane#8-13
P-TBK1
Lane#8-13
IRF3

Fig.1F

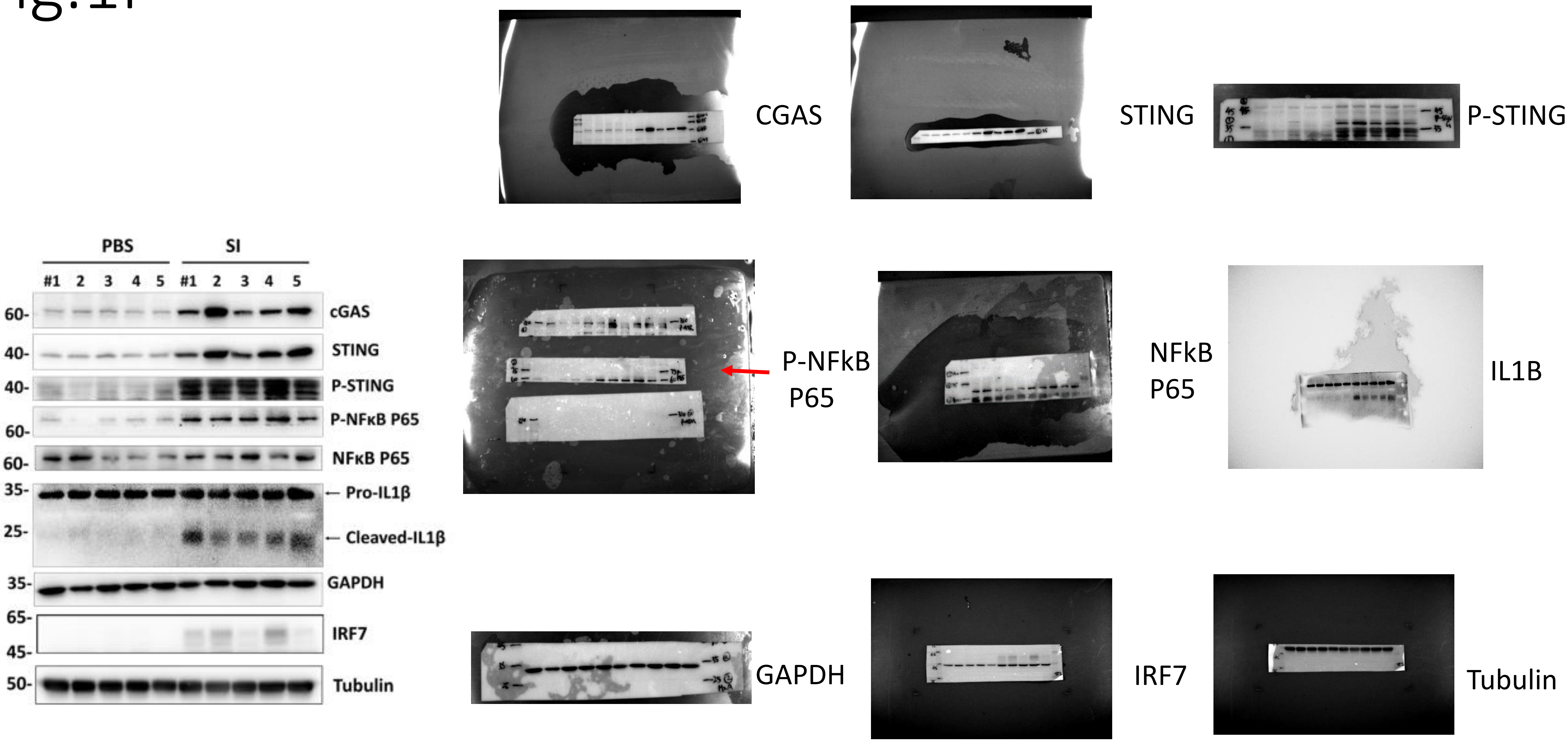
PBS
SI
#1 2 3 4 5 #1 2 3 4 5
60-
cGAS
40-
STING
40-
P-STING
60-
P-NFκB P65
60-
NFκB P65
35-
← Pro-IL1β
25-
← Cleaved-IL1β
35-
GAPDH
65-
45-
IRF7
50-
Tubulin
CGAS
STING
P-STING
P-NFkB
P65
NFkB
P65
IL1B
GAPDH
IRF7
Tubulin

Fig.1G

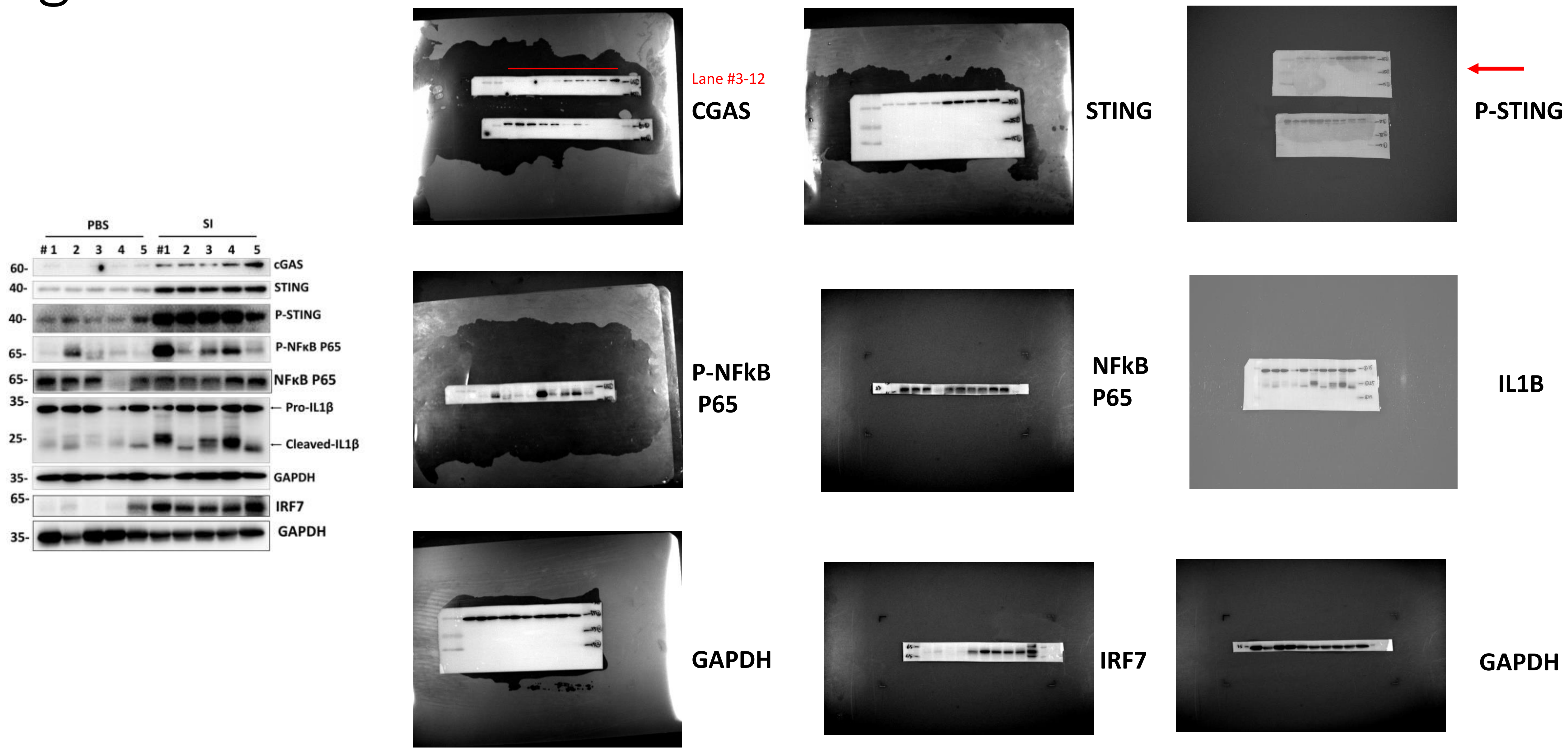
PBS
SI
#1 2 3 4 5 #1 2 3 4 5
60- cGAS
40- STING
40- P-STING
65- P-NFκB P65
65- NFκB P65
35- Pro-IL1β
25- Cleaved-IL1β
35- GAPDH
65- IRF7
35- GAPDH
Lane #3-12
CGAS
STING
P-STING
P-NFkB P65
NFkB P65
IL1B
GAPDH
IRF7
GAPDH

Fig.1 I

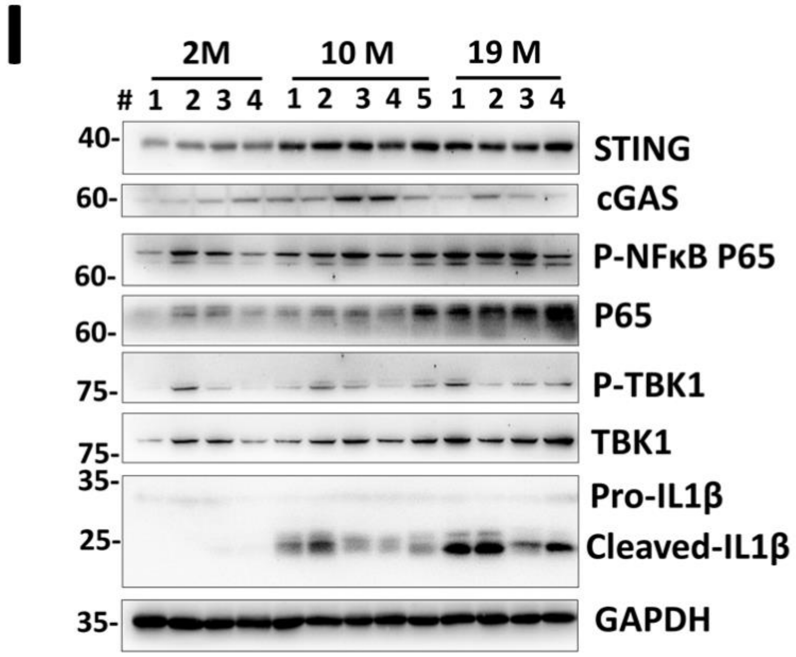
I
2M
10 M
19 M
1 2 3 4 1 2 3 4 5 1 2 3 4
40-
STING
60-
cGAS
P-NFκB P65
60-
P65
60-
P-TBK1
75-
TBK1
75-
35-
Pro-IL1β
25-
Cleaved-IL1β
35-
GAPDH

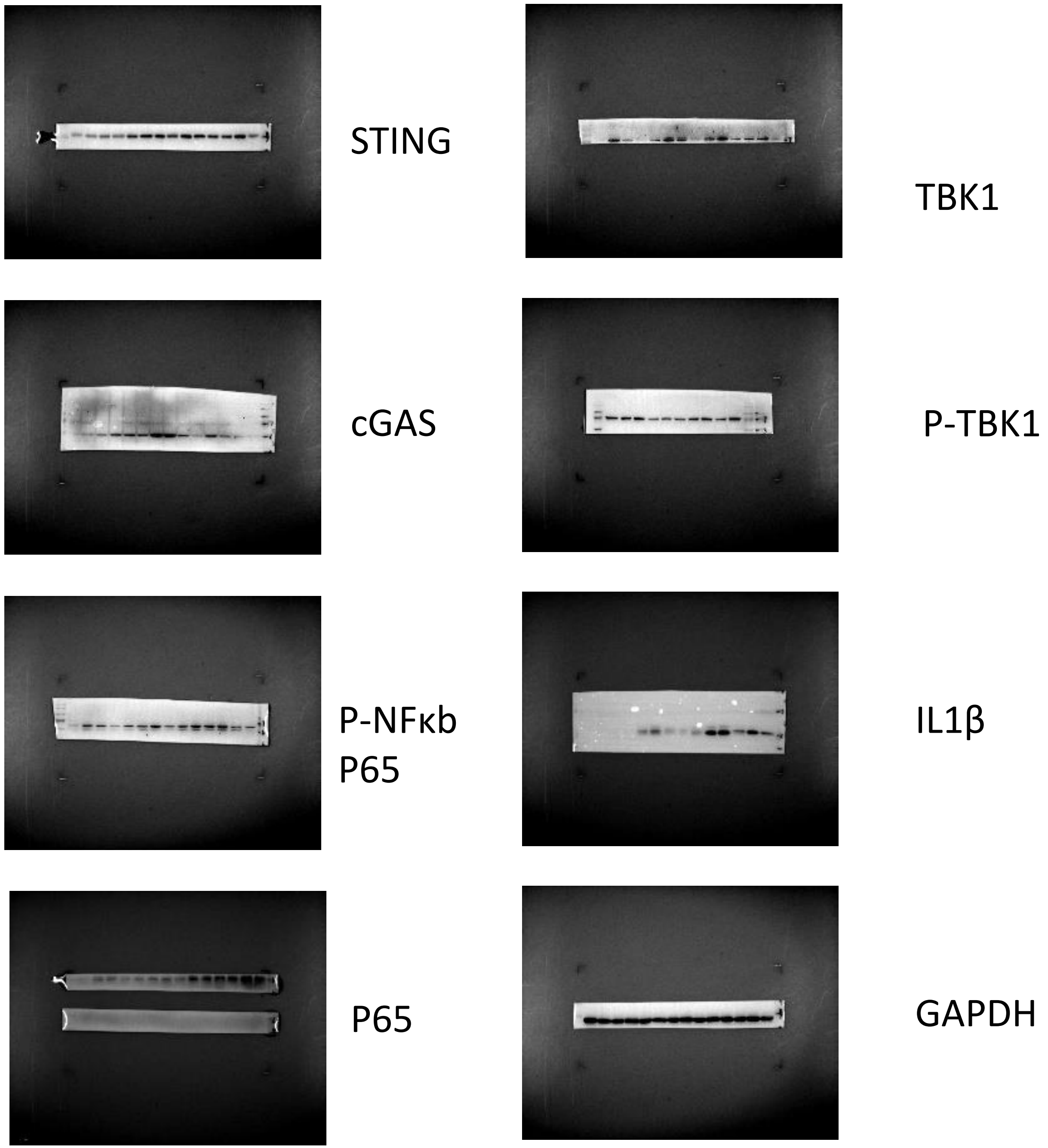
STING
TBK1
cGAS
P-TBK1
P-NFκb
P65
IL1β
P65
GAPDH

Fig.2C

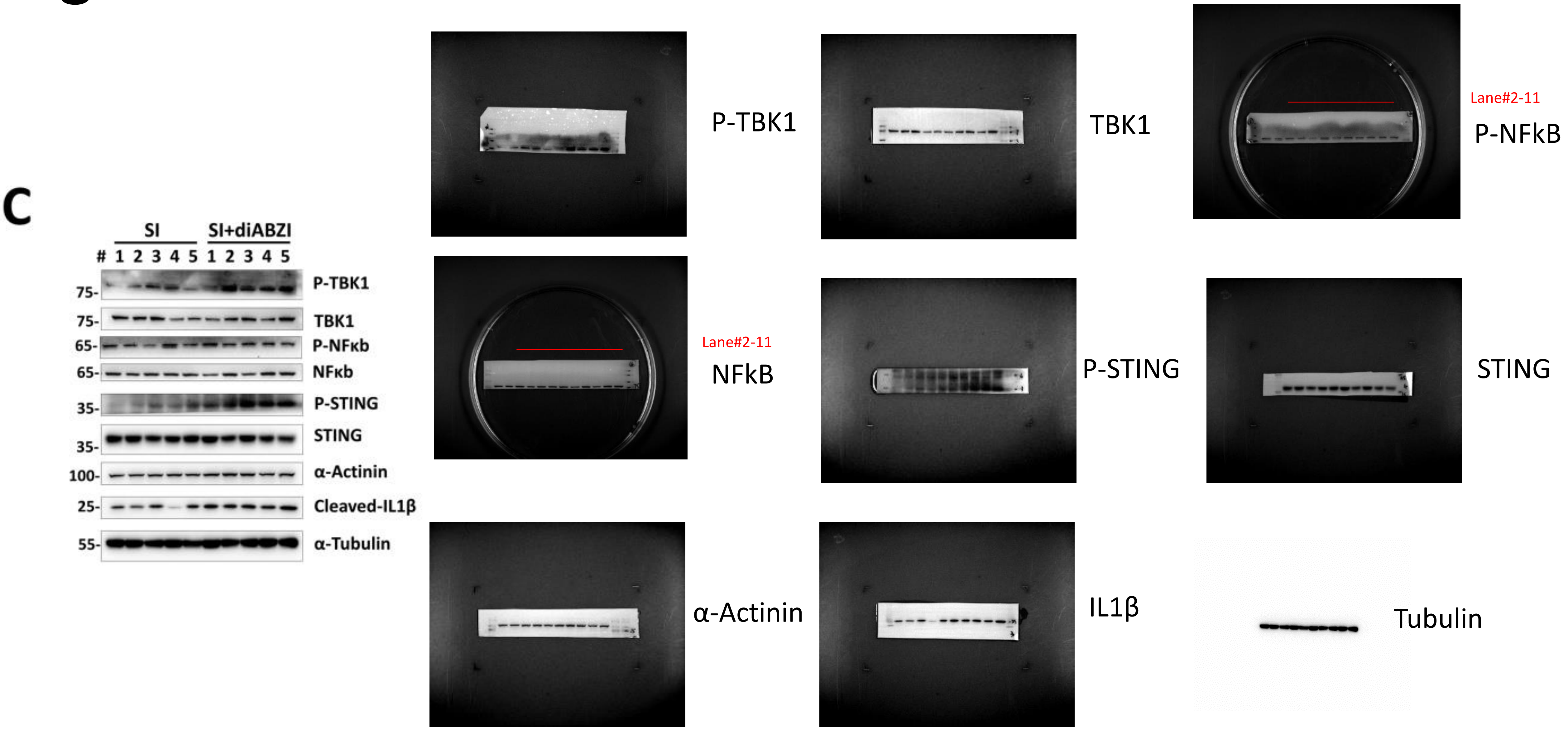
C
SI
SI+diABZI
1 2 3 4 5 1 2 3 4 5
75- P-TBK1
75- TBK1
65- P-NFκb
65- NFκb
35- P-STING
35- STING
100- α-Actinin
25- Cleaved-IL1β
55- α-Tubulin
P-TBK1
TBK1
Lane#2-11
P-NFkB
Lane#2-11
NFkB
P-STING
STING
α-Actinin
IL1β
Tubulin

Fig.2E

E

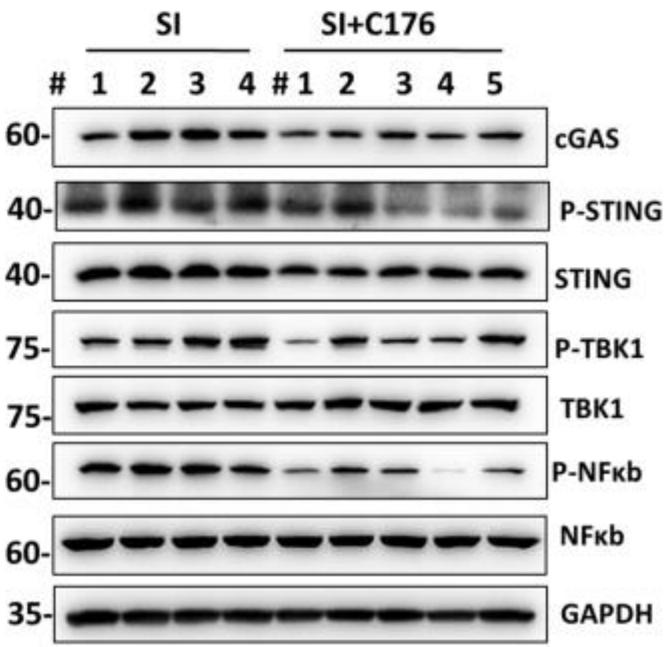

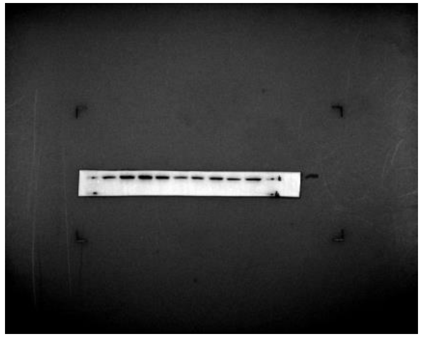

cGAS

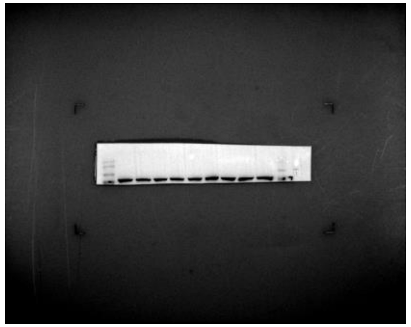

TBK1

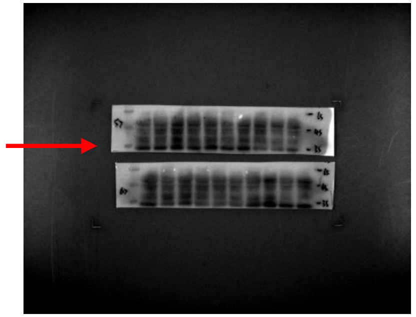

P-STING

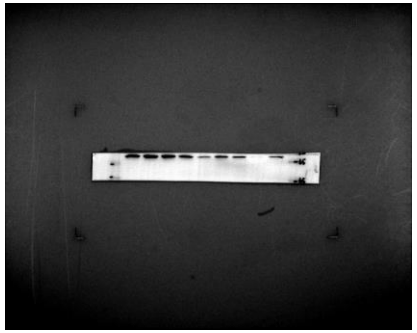

P-NFκb

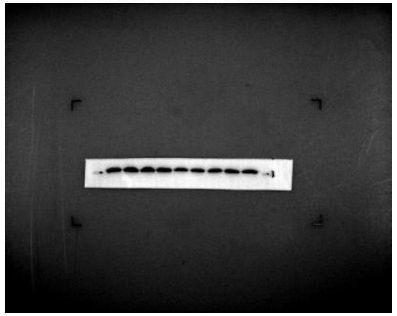

STING

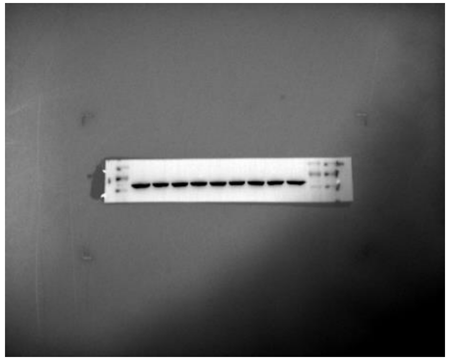

NFκb

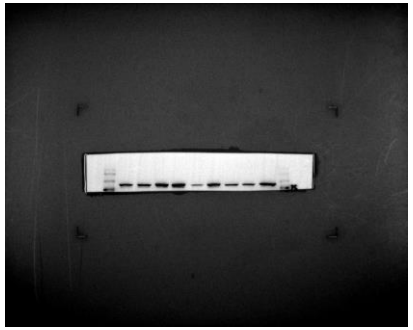

P-TBK1

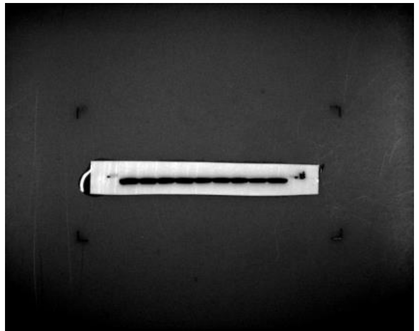

GAPDH

Fig.2J

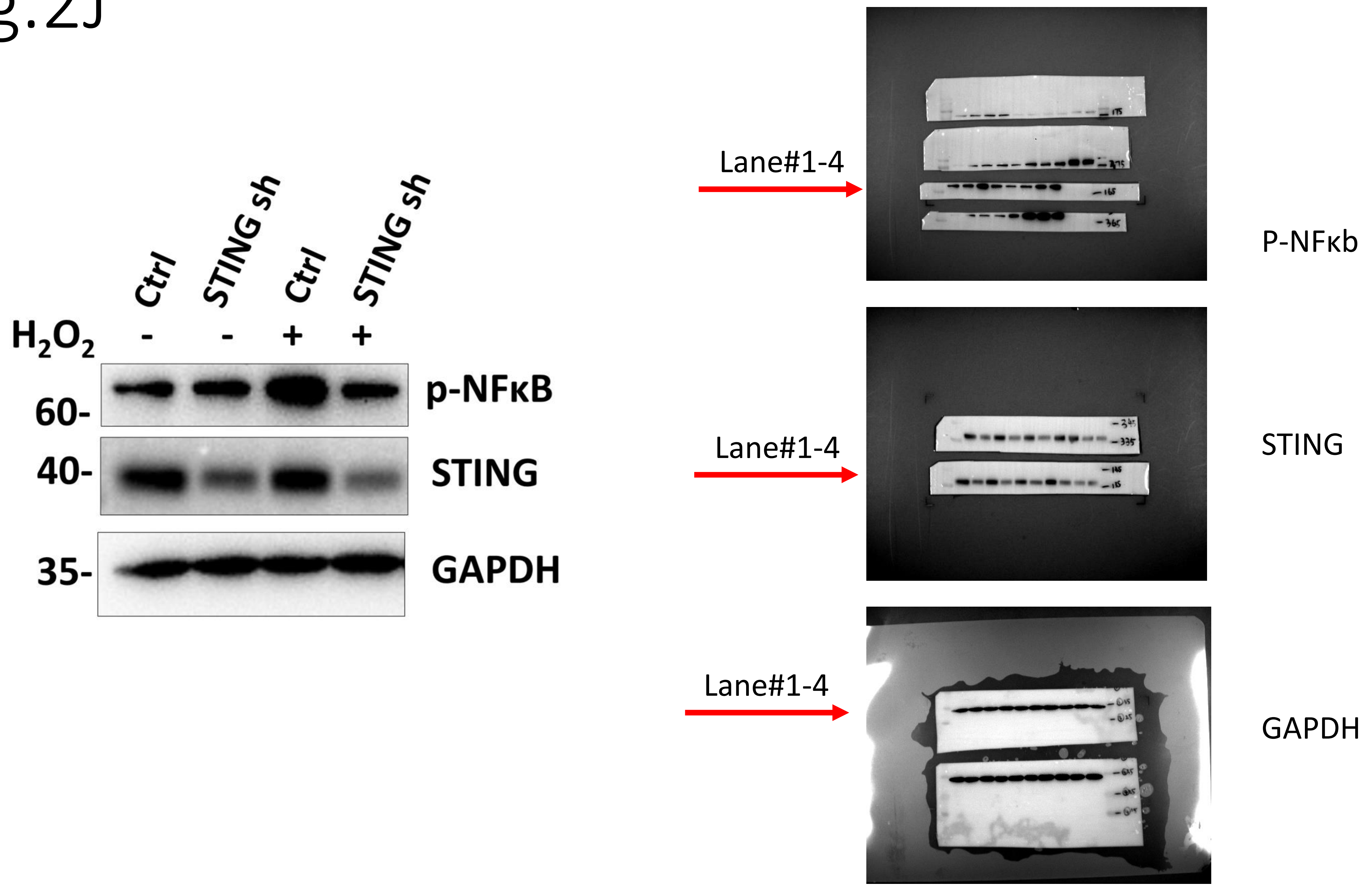

Ctrl
STING sh
Ctrl
STING sh
H_2O_2 - - + +
60-
40-
35-
p-NFκB
STING
GAPDH
Lane#1-4
Lane#1-4
Lane#1-4
P-NFκb
STING
GAPDH

Fig.3D

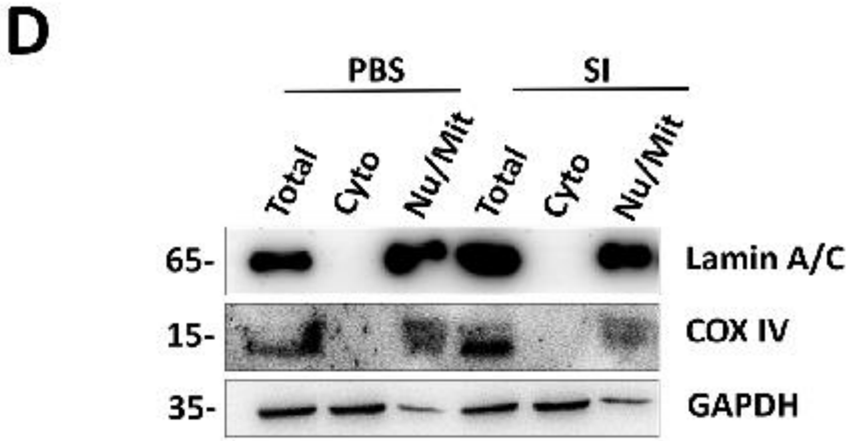

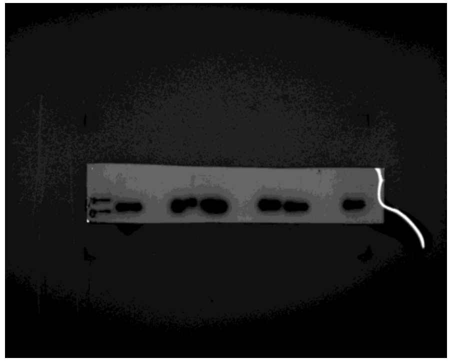

Lamin A/C

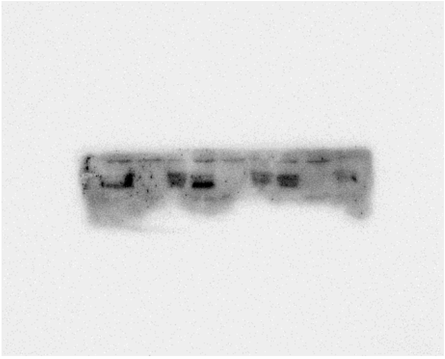

Long exposure

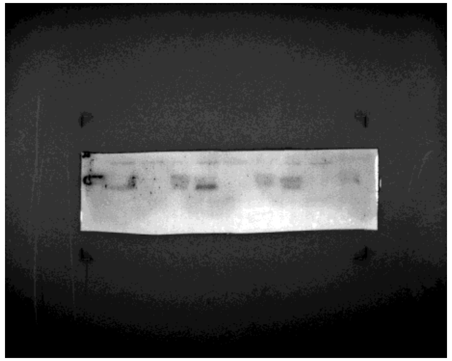

COX IV

Short exposure

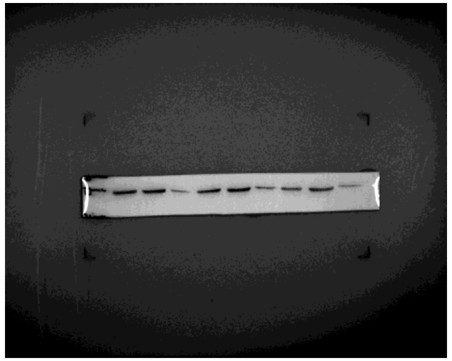

GAPDH

Fig.3H

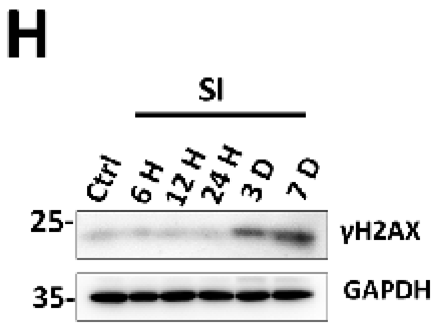

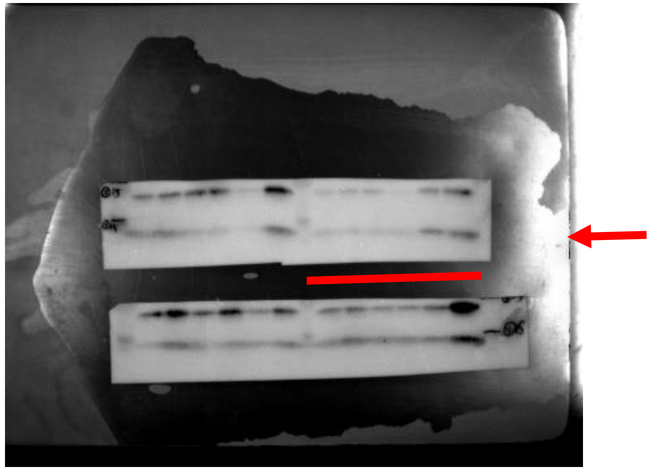

γ-H2AX

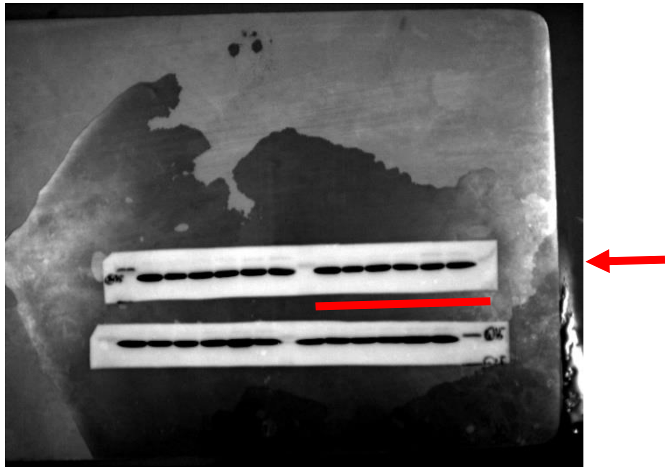

GAPDH

Fig.3I

I

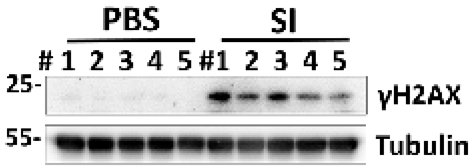

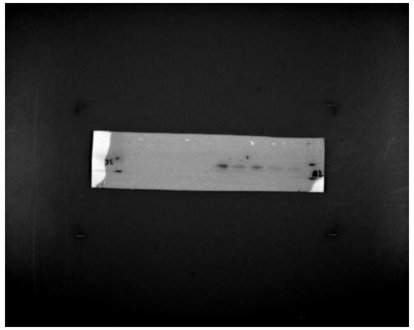

γH2AX

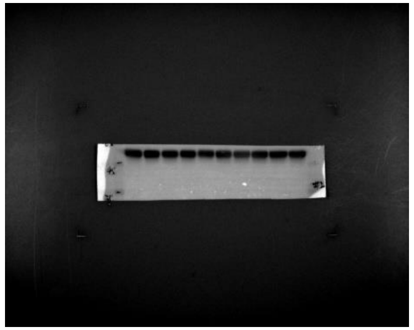

β-tubulin

Fig.3 J

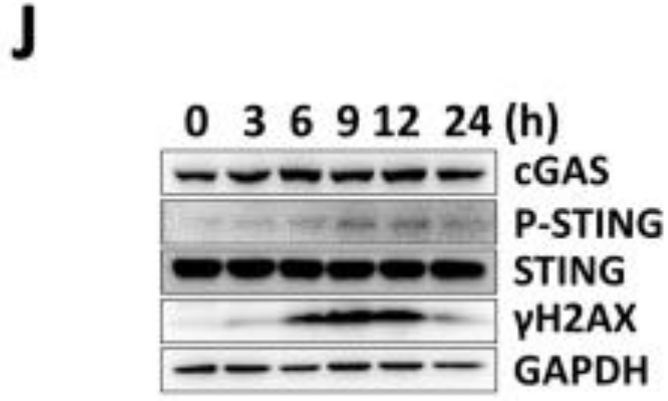

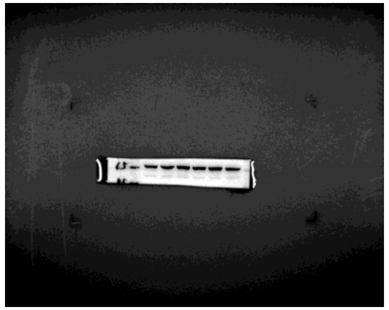

CGAS

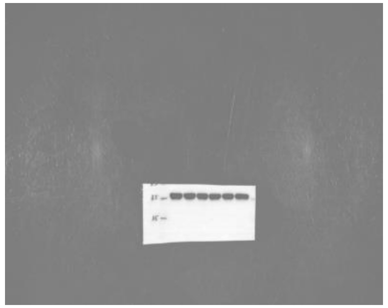

STING

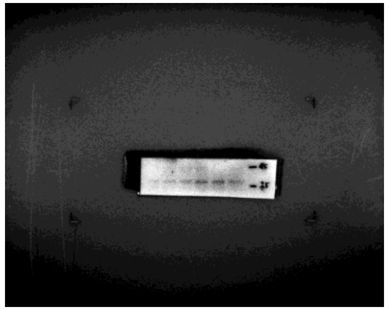

P-STING

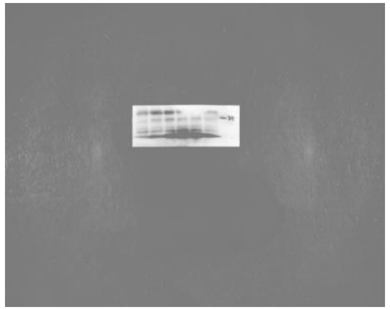

rH2AX

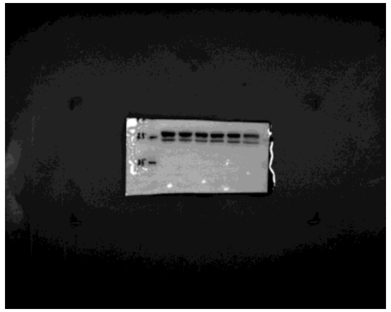

GAPDH

Fig.3M

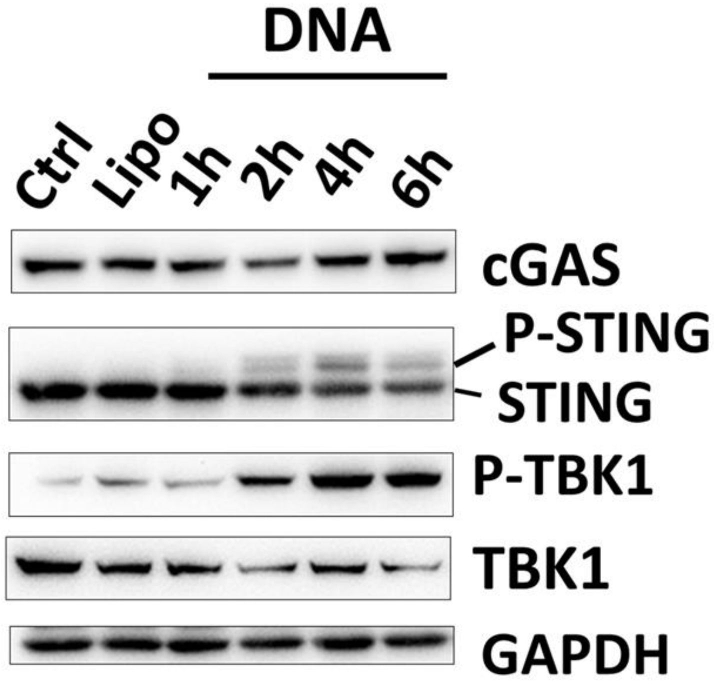

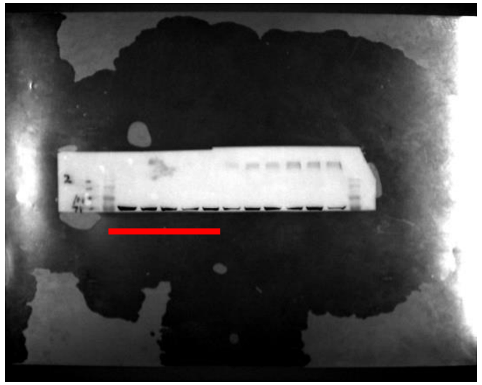

Lane#1-6
TBK1

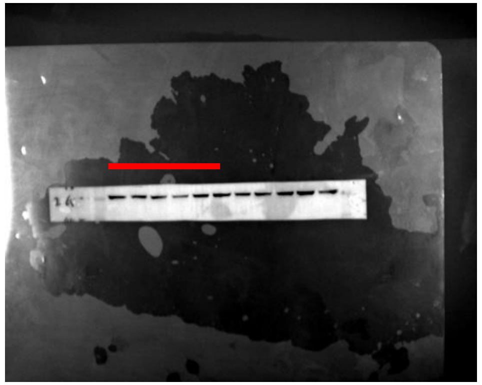

Lane#1-6
cGAS

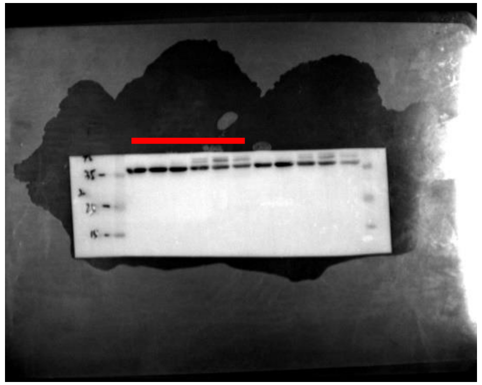

Lane#1-6
P-STING
&STING

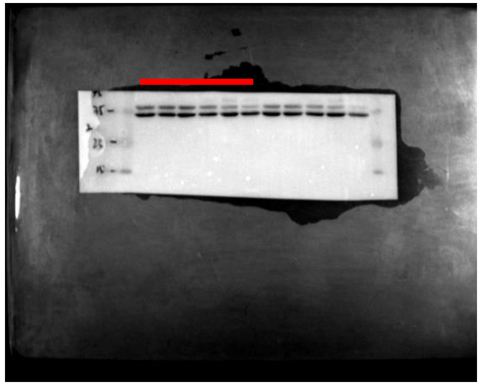

Lane#1-6
GAPDH

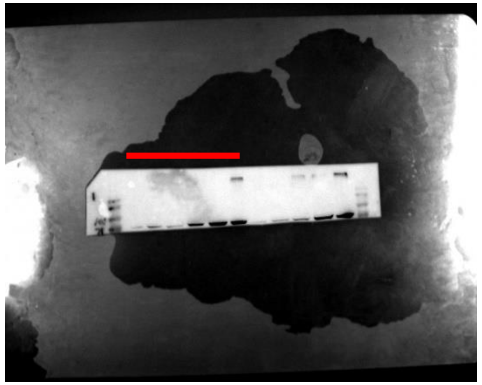

Lane#1-6
P-TBK1

Fig.3 N

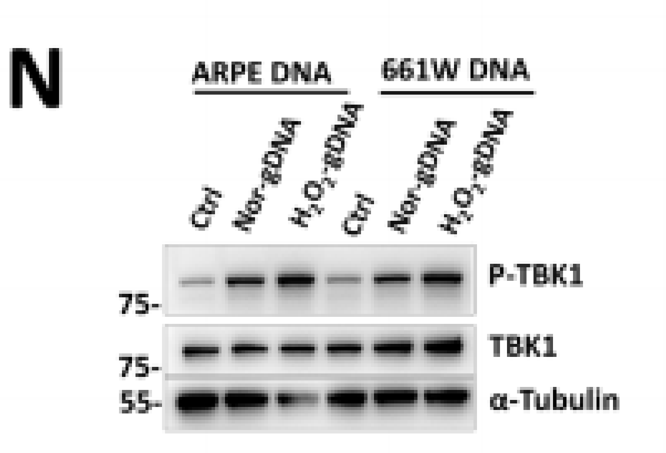

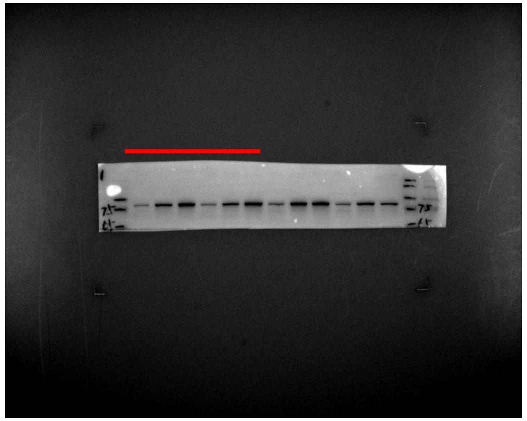

Lane #1-6

P-TBK1

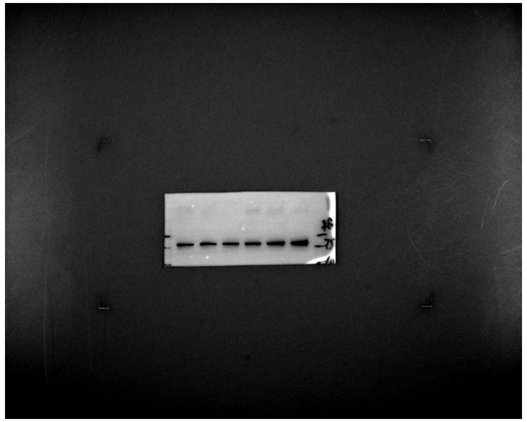

TBK1

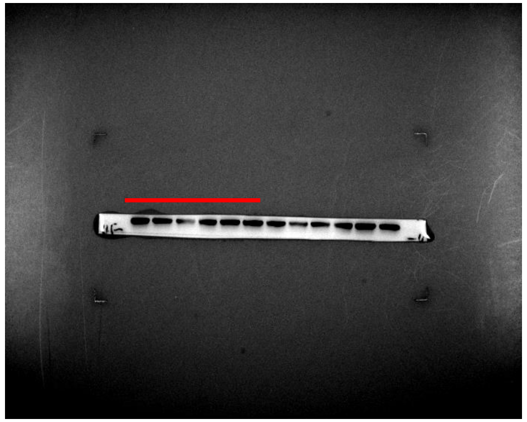

Lane #1-6

a-Tubulin

Fig.4C

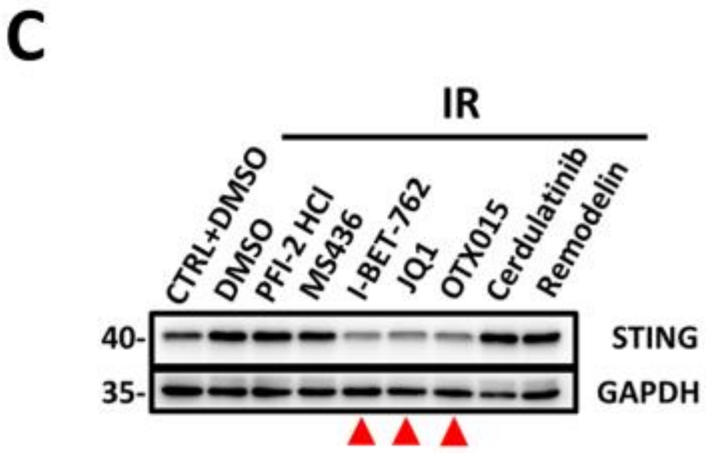

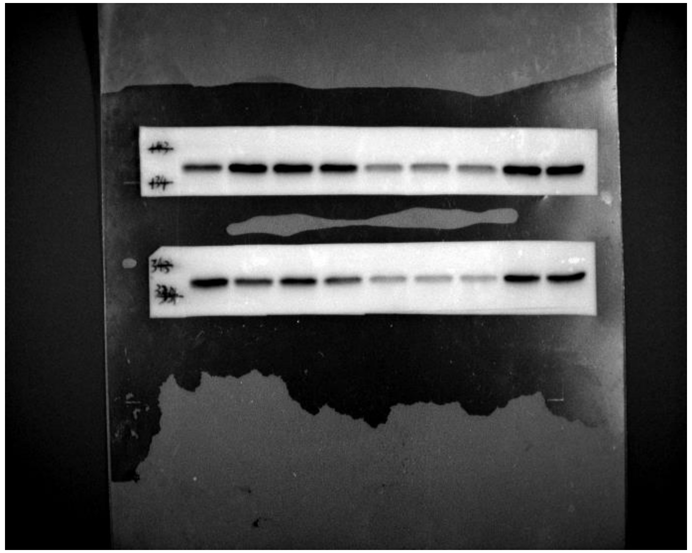

STING

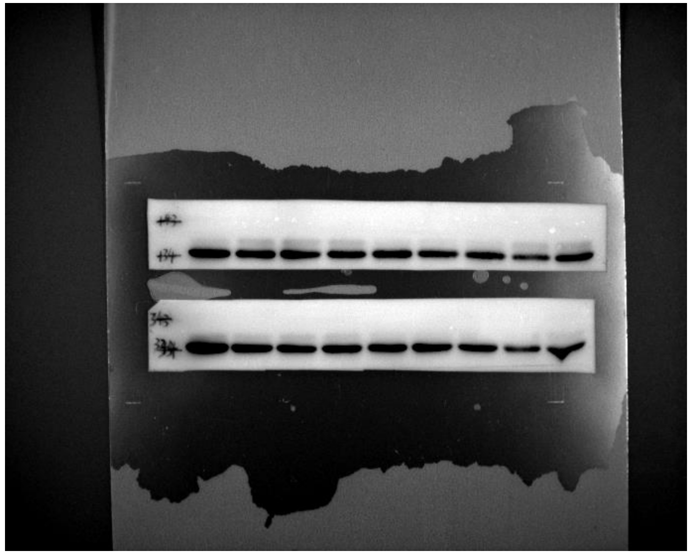

GAPDH

Fig.4E

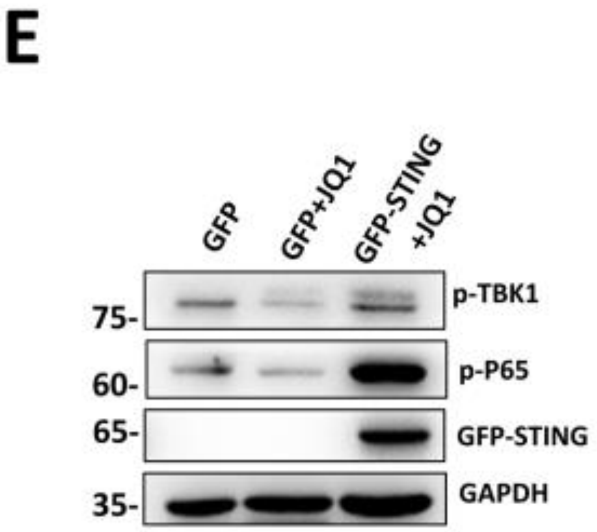

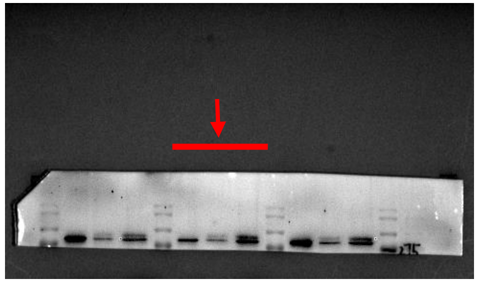

P-TBK1

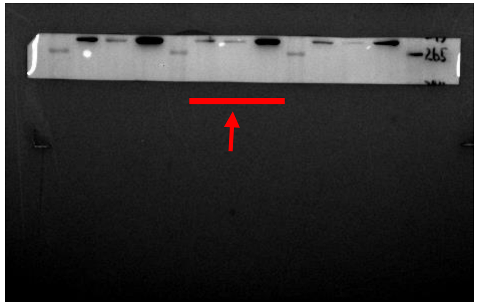

P-P65

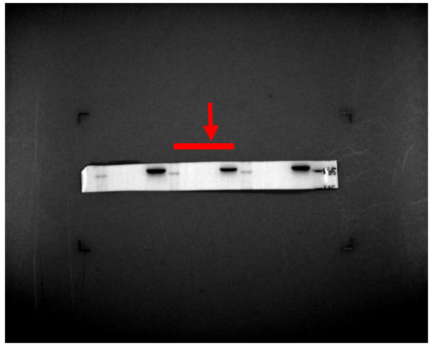

GFP-STING

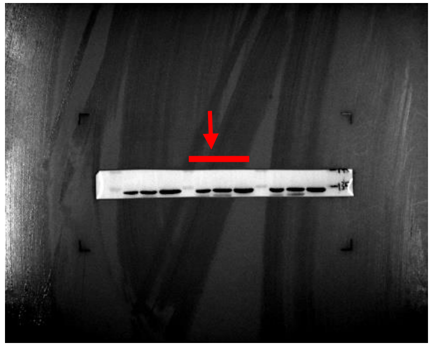

GAPDH

Fig.5F

F

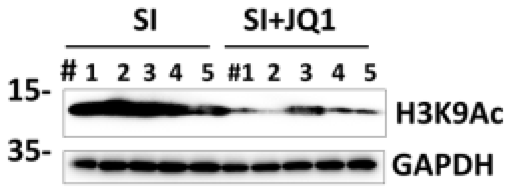

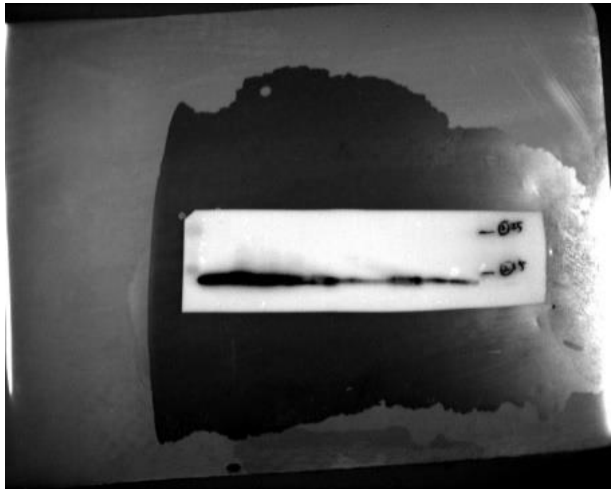

H3K9AC

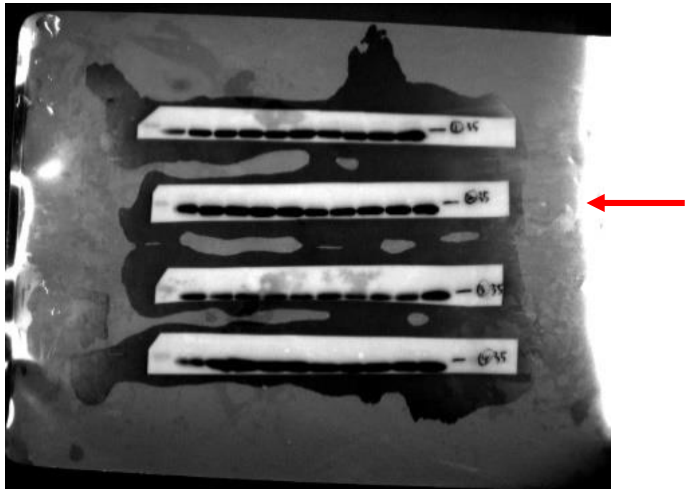

GAPDH

Fig.6B

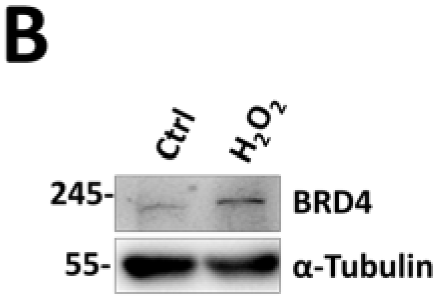

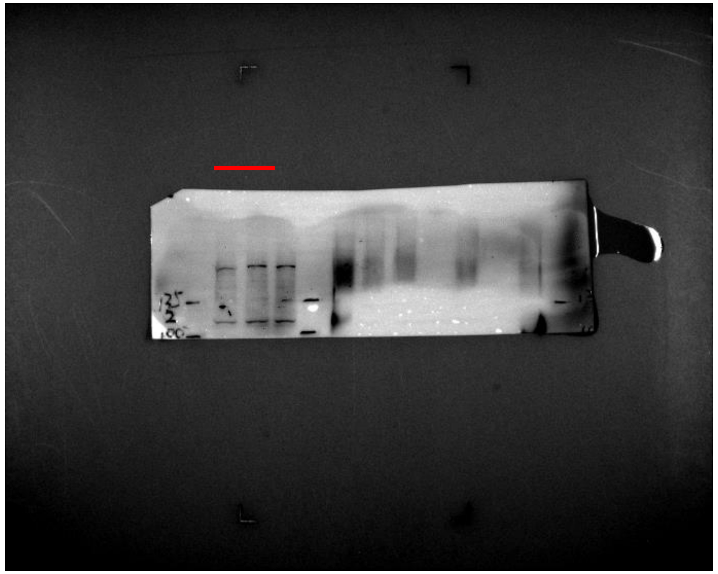

Lane#1-2

BRD4

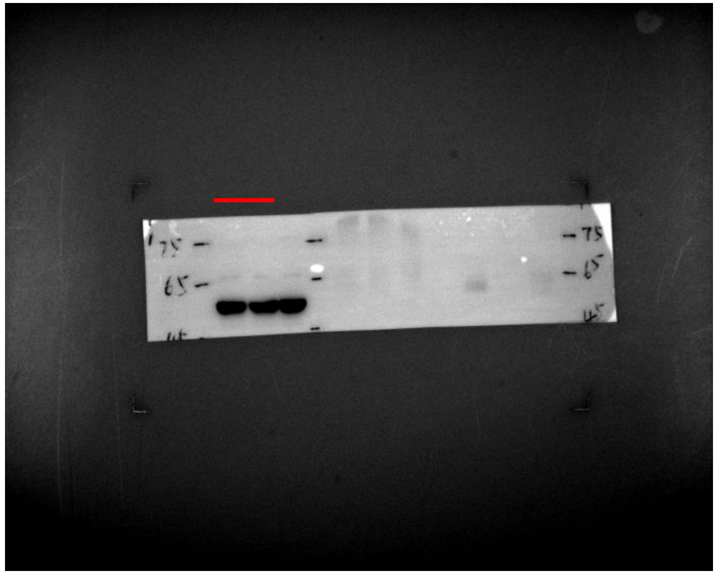

Lane#1-2

α-Tubulin

Fig.6C

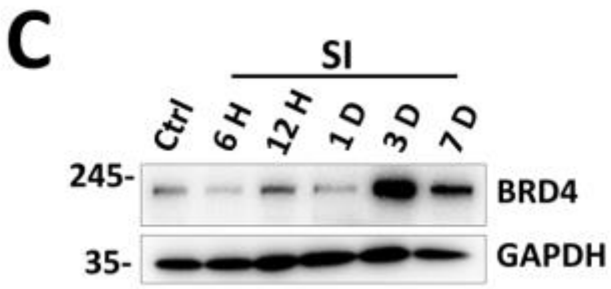

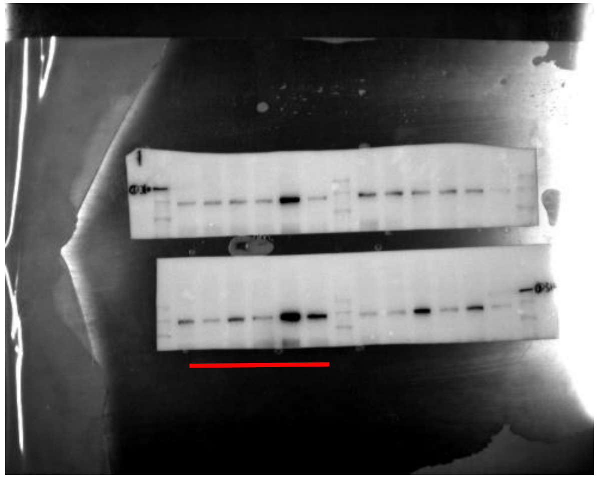

Lane#1-6

BRD4

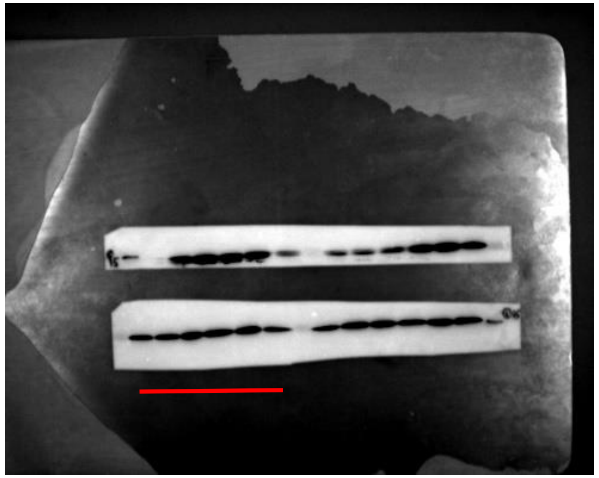

Lane#1-6

GAPDH

Fig.6D

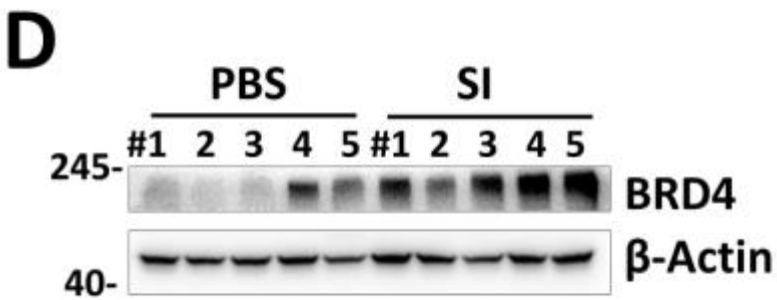

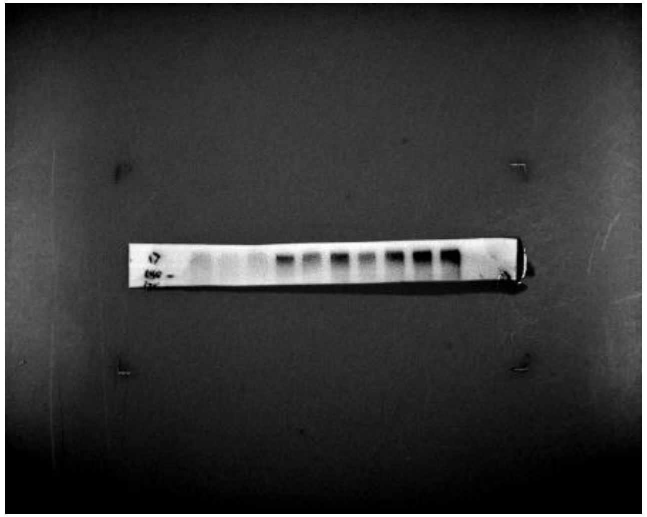

BRD4

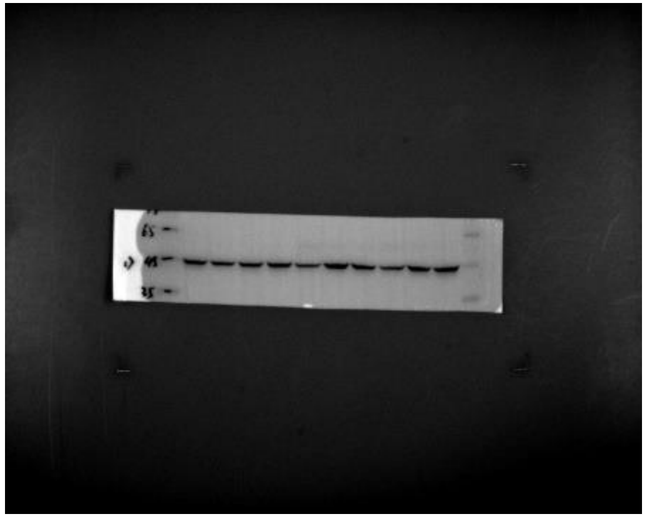

β-Actin

Fig.6 F

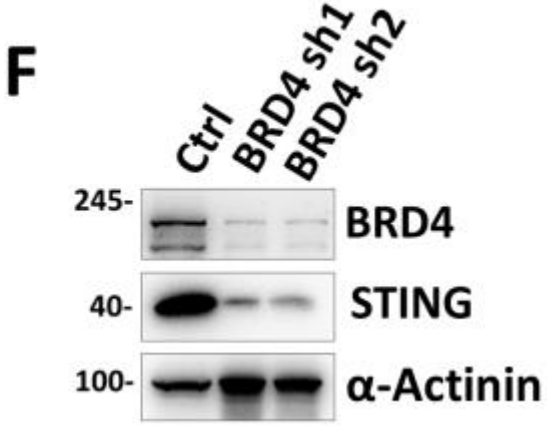

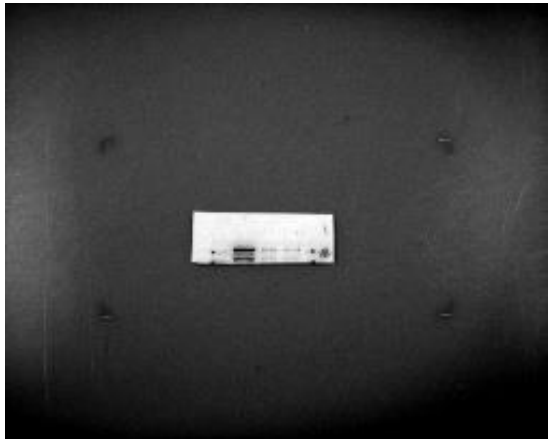

BRD4

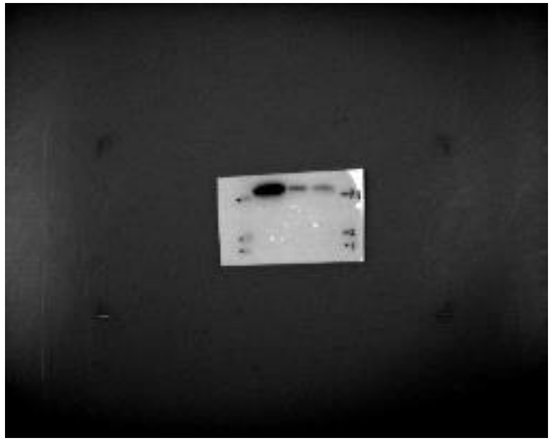

STING

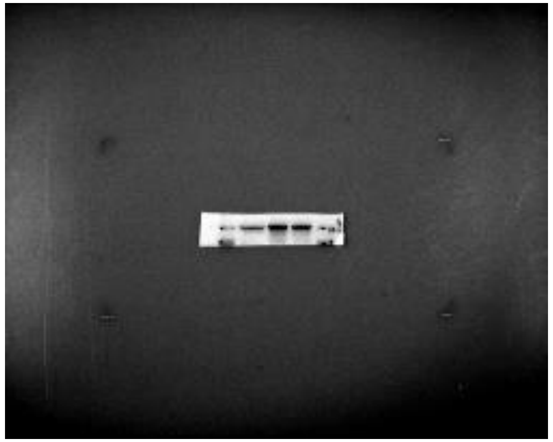

α-Actinin

Fig.7A

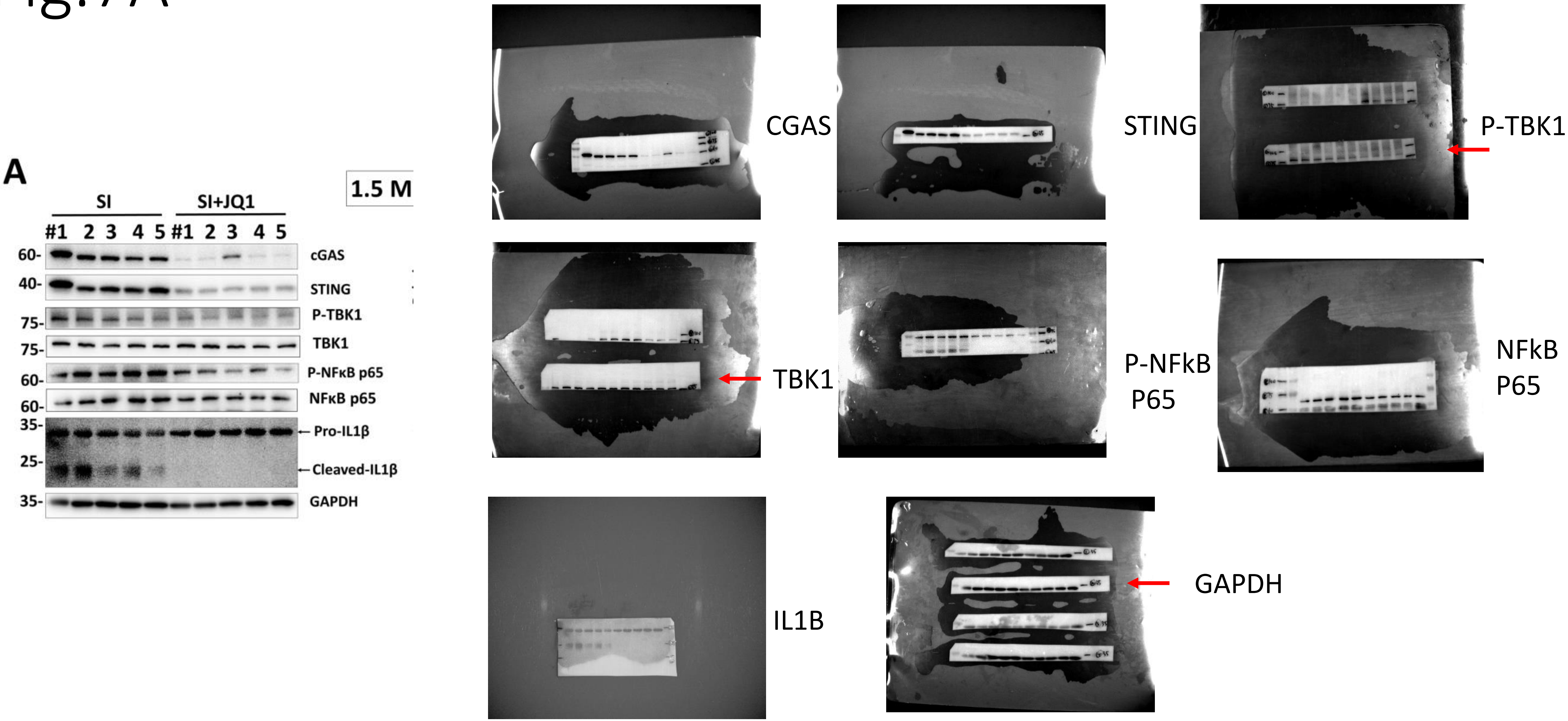
A
1.5 M
SI
SI+JQ1
#1 2 3 4 5 #1 2 3 4 5
60-
40-
75-
75-
60-
60-
35-
25-
35-
cGAS
STING
P-TBK1
TBK1
P-NFκB p65
NFκB p65
Pro-IL1β
Cleaved-IL1β
GAPDH
CGAS
STING
P-TBK1
TBK1
P-NFkB
P65
NFkB
P65
IL1B
GAPDH

Fig.7B

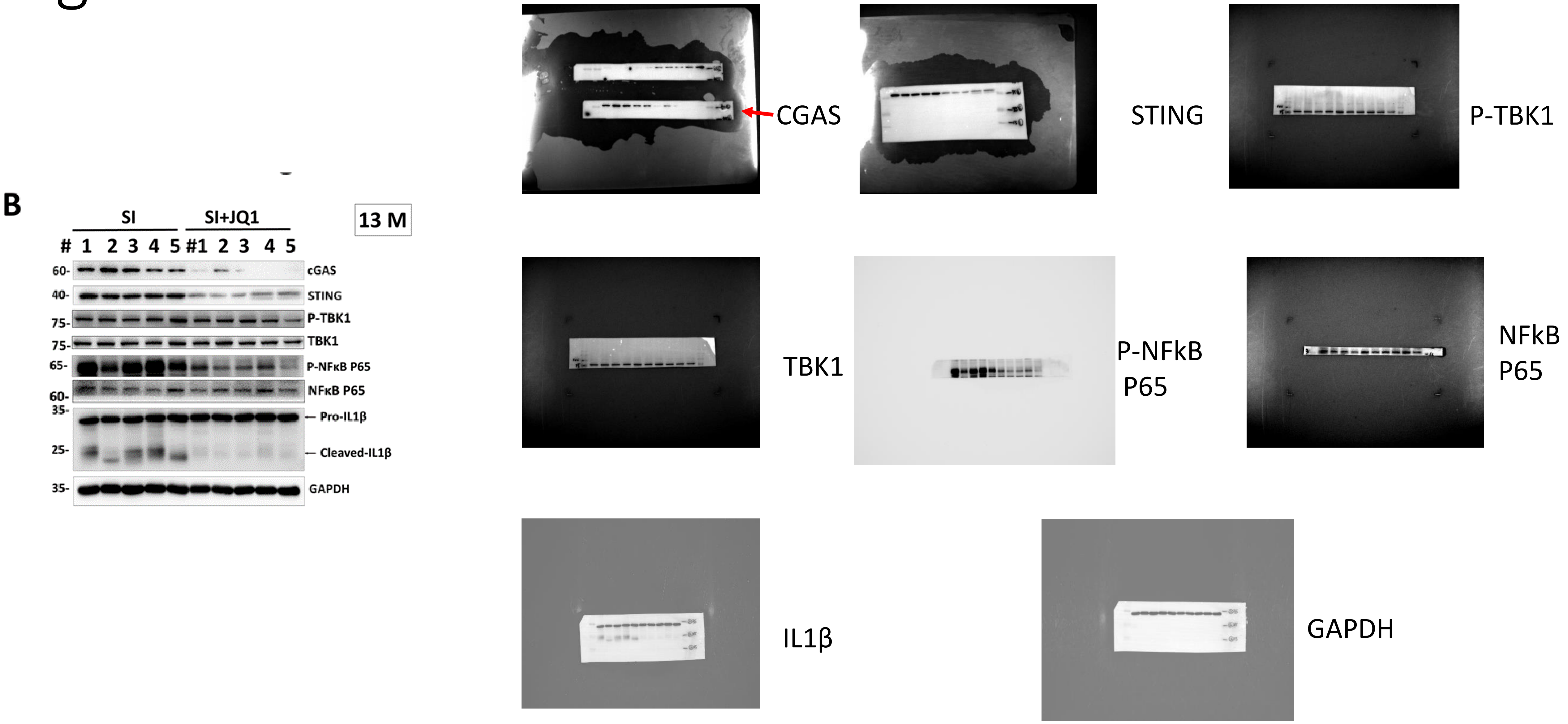
B
SI
SI+JQ1
13 M
1 2 3 4 5 #1 2 3 4 5
60-
cGAS
40-
STING
75-
P-TBK1
75-
TBK1
65-
P-NFκB P65
60-
NFκB P65
35-
Pro-IL1β
25-
Cleaved-IL1β
35-
GAPDH
CGAS
STING
P-TBK1
TBK1
P-NFkB P65
NFkB P65
IL1β
GAPDH

Fig.7D

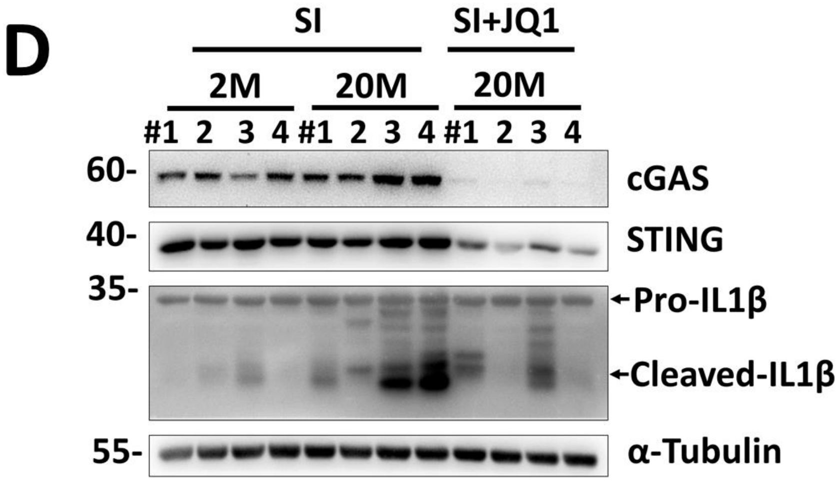

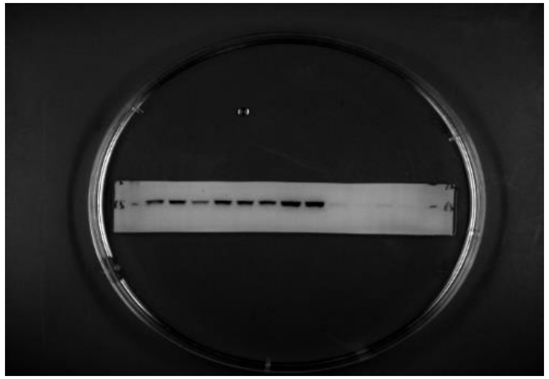

cGAS

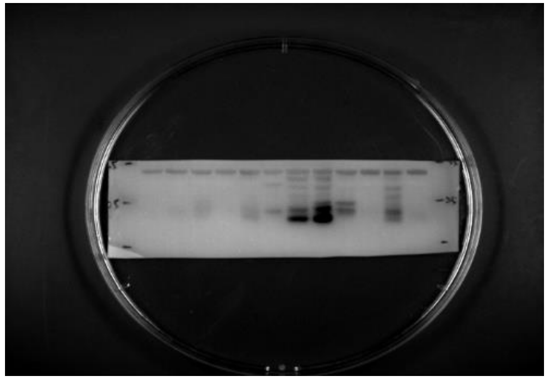

IL1β

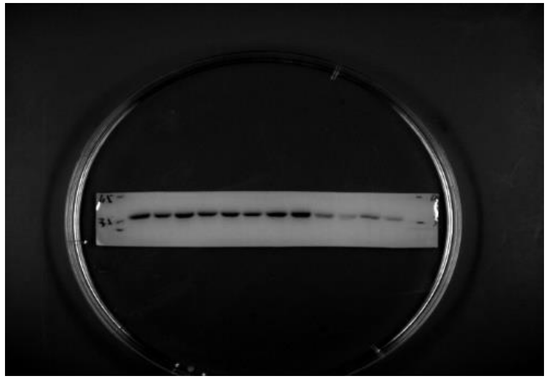

STING

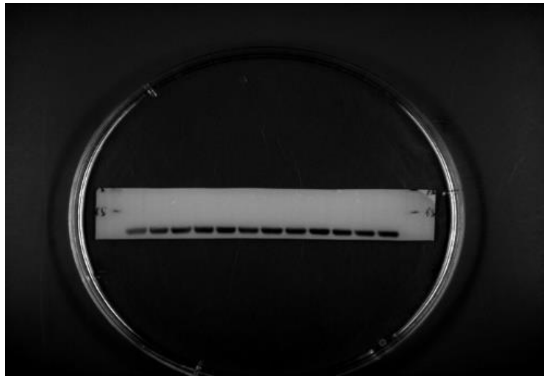

α-Tubulin

Fig.8B

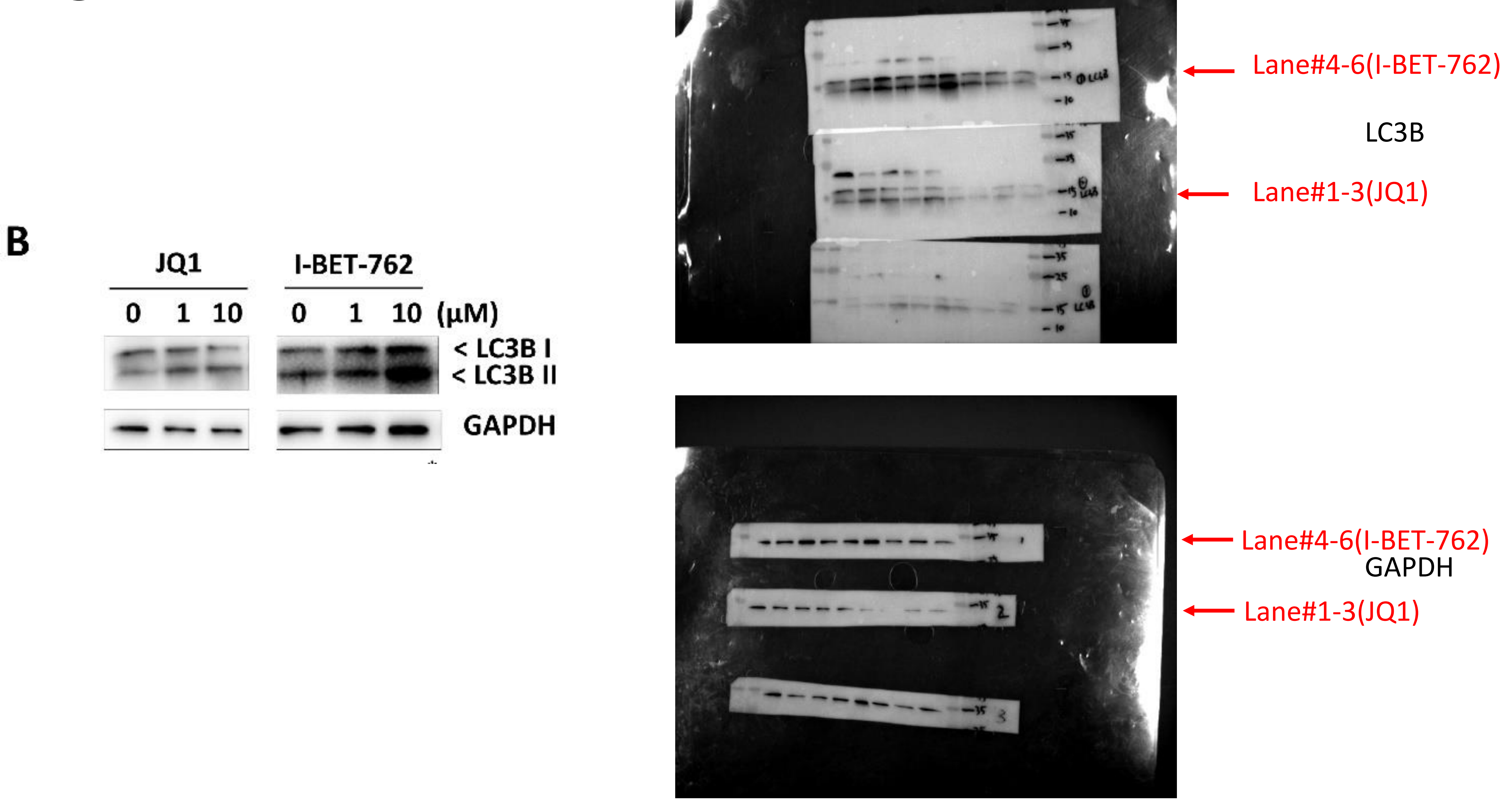
B
JQ1
I-BET-762
0 1 10
0 1 10 (μM)
< LC3B I
< LC3B II
GAPDH
Lane#4-6(I-BET-762)
LC3B
Lane#1-3(JQ1)
Lane#4-6(I-BET-762)
GAPDH
Lane#1-3(JQ1)

Fig.8 C

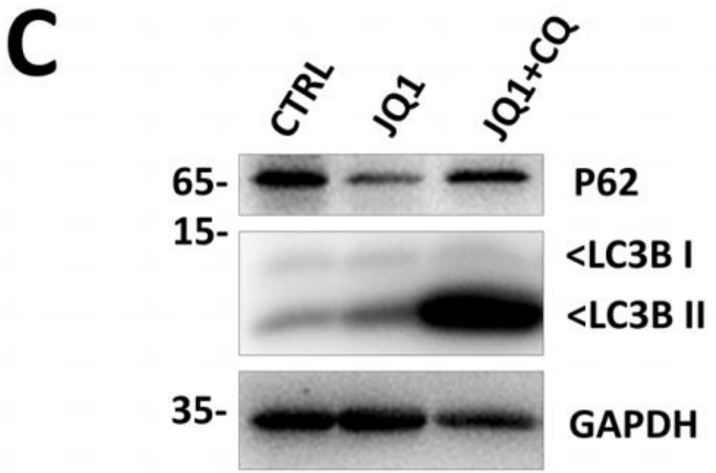

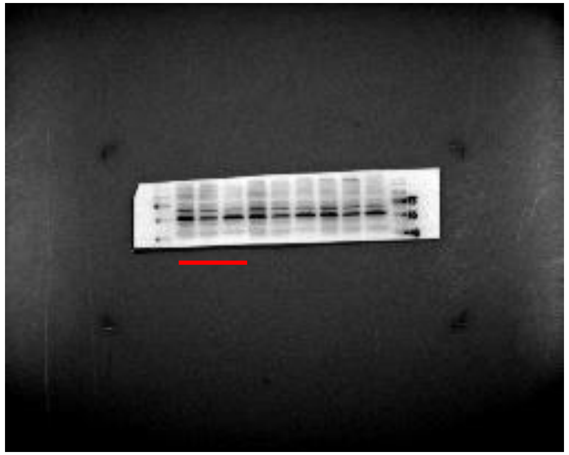

Lane#1-3

P62

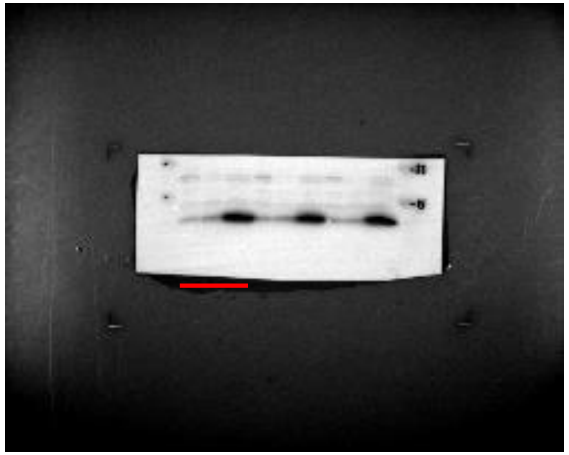

Lane#1-3

lc3b

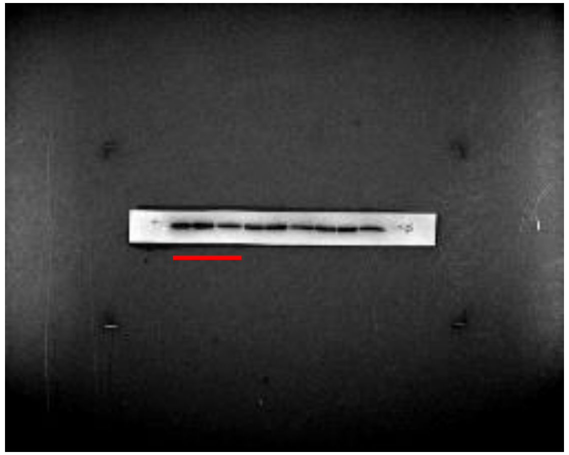

Lane#1-3

GAPDH

Fig.8D

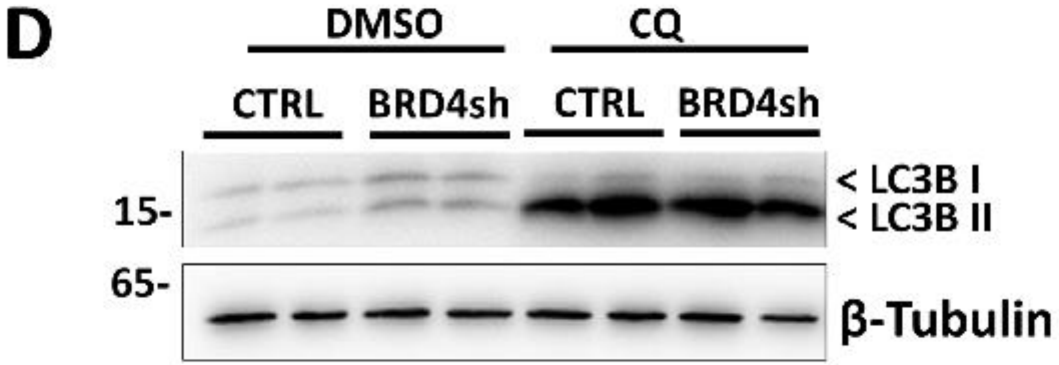

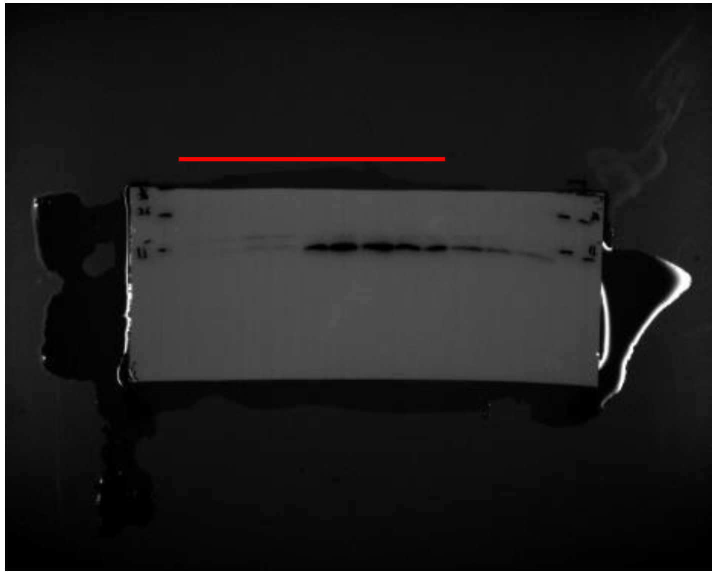

Lane#1-8

LC3B

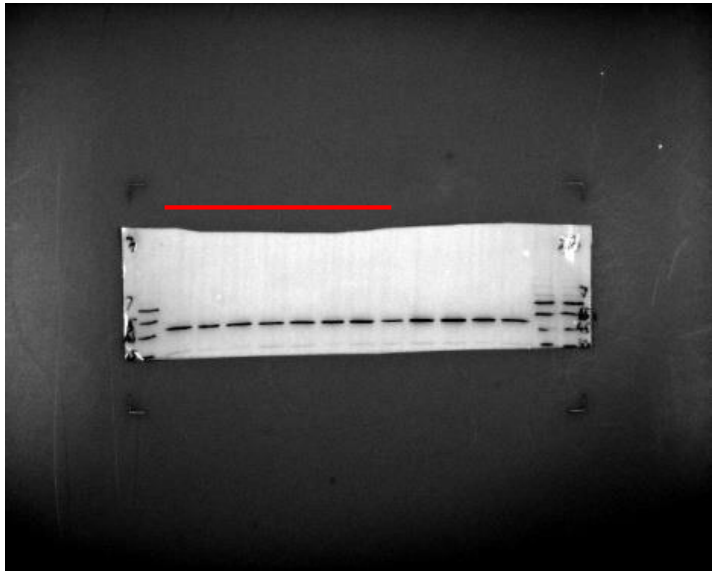

Lane#1-8

β-Tubulin

Fig.S1

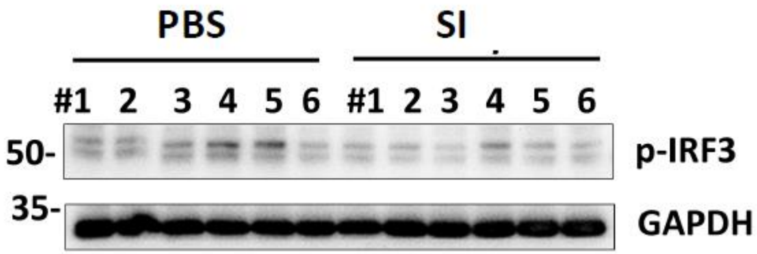

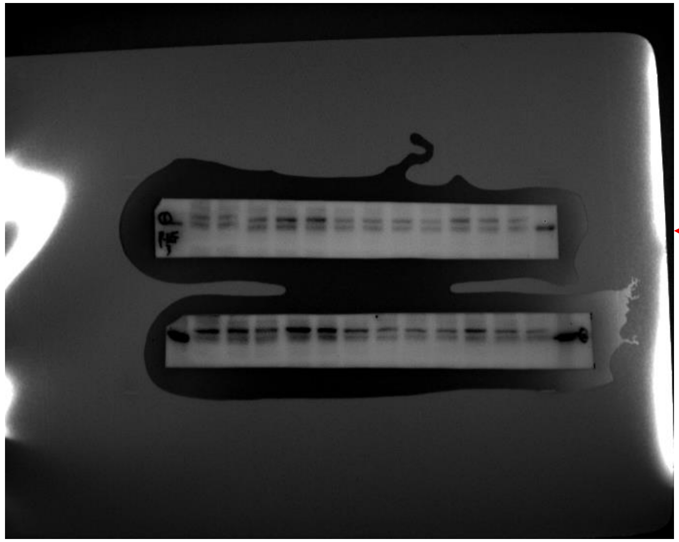

P-IRF3

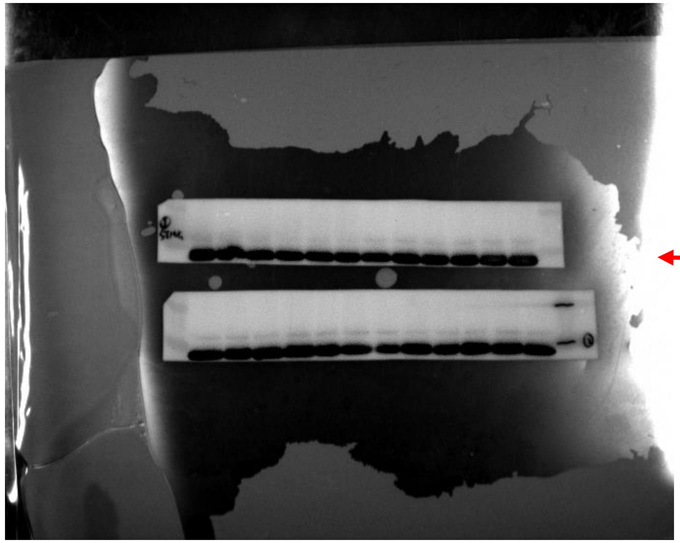

GAPDH

Fig.S2

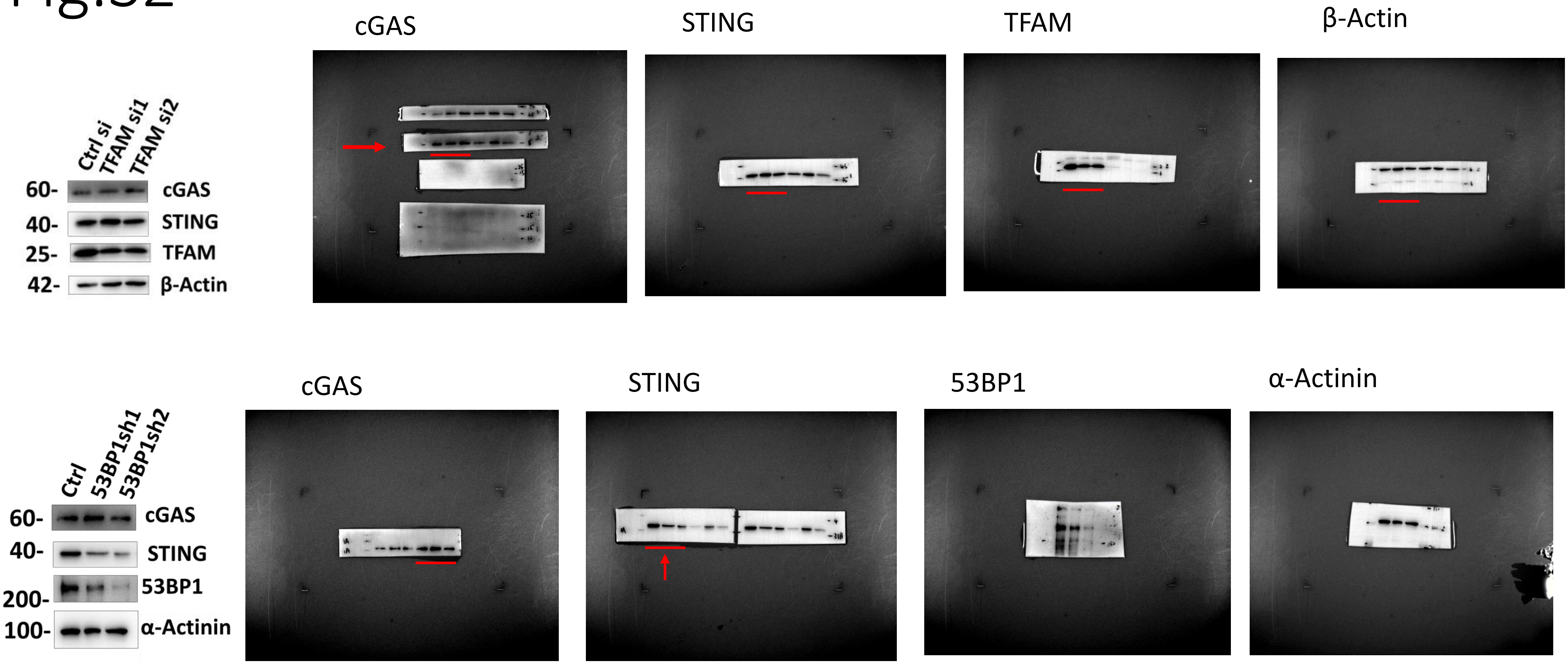

cGAS
STING
TFAM
β-Actin
Ctrl si
TFAM si1
TFAM si2
60-
cGAS
40-
STING
25-
TFAM
42-
β-Actin
cGAS
STING
53BP1
α-Actinin
Ctrl
53BP1sh1
53BP1sh2
60-
cGAS
40-
STING
53BP1
200-
100-
α-Actinin
