# Supplementary material for: Inhibition of cGAS-STING by JQ1 alleviates oxidative stress-induced retina inflammation and degeneration
Source: Cell Death Differ. 2022 Mar 28;29(9):1816–33. doi: 10.1038/s41418-022-00967-4 (PMC9433402; doi:10.1038/s41418-022-00967-4)
Supplement: Supplementary file 5 — Supplementary figure and table legends [file 41418_2022_967_MOESM5_ESM.docx]

**Supplementary Fig. 1 IRF3 was not activated in mouse retina upon SI injection.**

**A** WB analysis for p-IRF3 in mouse retina with PBS or SI injection.

**B** quantification of the results in of A

**C** qRT-PCR analysis of the relative retina *IRF3* RNA levels in PBS- or SI-injected mice. P-values were calculated using a two-tailed unpaired Student’s t test. *, P < 0.05 and **, P < 0.005.

**Supplementary Fig. 2. Confirmation for the knockdown of TFAM and 53BP1 in ARPE cells**

A-B. Left: qRT-PCR shows knockdown of TFAM or 53BP1 by two independent sets of siRNA or shRNA, respectively. Right: WB analysis shows decreased TFAM or 53BP1 after the indicated knockdown conditions. Note that cGAS and STING proteins levels are not significantly altered.

**Supplementary Fig. 3. JQ1 does not alter eye morphology, retina structure or retinal cell viability.**

PBS or JQ1 (50 mg/kg) was I.P injected daily, and the observation was made up to 6 days post- injection. For each treatment, 4 mice were investigated per group.

**A** Fundus photography and fluorescein angiography show no gross eye morphology change after JQ1 injection.

**B** TUNEL labeling indicates that JQ1 treatment does not cause retinal cell death. The DNase I-treated slide served as positive control. Scale bar: 200 μM.

**C-D** IF analysis showed that the gross morphology of photoreceptors (C) and optic nerve system (D) were not altered by JQ1 injection. The photoreceptors and optic nerve were visualized by recoverin and GFAP staining, respectively. Scale bar: 200 μM.

**Supplementary Fig. 4. Original WB results in the manuscript**

All uncropped original WB results are shown. The membranes were first captured under the white light to obtain the whole image containing the molecular weight, and then the luminescence image were obtained under various exposure time. The luminescent and bright images were merged and shown for each blots.

**Supplementary video 1**

Cy3-DNA was transfected into ARPE LC3-GFP cells, and 6h after transfection, cells were treated with DMSO or JQ1 (1 or 10 μM). Recording started at 2 h after addition of JQ1 and lasted 24 h with 10 min intervals.

**Table 1 Key reagents and resources used in this study.**

**Table 2 Primers used for qRT-PCR, qCHIP, MNase assay and mitochondria and nuclear DNA detection**
