# Supplementary material for: Inhibition of cGAS-STING by JQ1 alleviates oxidative stress-induced retina inflammation and degeneration
Source: Cell Death Differ. 2022 Mar 28;29(9):1816–33. doi: 10.1038/s41418-022-00967-4 (PMC9433402; doi:10.1038/s41418-022-00967-4)
Supplement: Supplementary file 7 — Revised Bullet Points [file 41418_2022_967_MOESM7_ESM.docx]

**Bullet Points**

STING transcription and chromatin accessibility increases in macular retina of dry AMD patients;

Cytosolic DNA leakage and cGAS-STING activation are detected in oxidative stress-induced retina degeneration

Oxidative stress increases BRD4 expression, BRD4 inhibition represses cGAS-STING and ameliorates retina degeneration and inflammation

DNA from H_2_O_2_-treated cell causes enhanced inflammation in cytosol, BRD4 inhibition promotes cytosolic DNA autophagy hence inflammation repression.
